# Neuroprotective effect and possible mechanism of edaravone in rat models of spinal cord injury: a systematic review and network meta-analysis

**DOI:** 10.3389/fphar.2025.1538879

**Published:** 2025-04-07

**Authors:** Long-yun Zhou, Xiao-bo Wang, Xu-qing Chen, Ran Li, Bin-bin Yu, Meng-xiao Pan, Lu Fang, Jian Li, Xue-jun Cui, Min Yao, Xiao Lu

**Affiliations:** ^1^ Department of Rehabilitation Medicine, The First Affiliated Hospital with Nanjing Medical University, Nanjing, China; ^2^ Spine Disease Institute, Longhua Hospital, Shanghai University of Traditional Chinese Medicine, Shanghai, China; ^3^ Key Laboratory of Theory and Therapy of Muscles and Bones, Ministry of Education, Shanghai University of Traditional Chinese Medicine, Shanghai, China; ^4^ Department of Otolaryngology, Jiangsu Province Hospital of Chinese Medicine, Affiliated Hospital of Nanjing University of Chinese Medicine, Nanjing, Jiangsu, China; ^5^ Department of Rehabilitation Medicine, Traditional Chinese Medicine Hospital of LuAn, Luan, China

**Keywords:** edaravone, spinal cord injury, systematic review, neuroprotective effect, neurological function, action mechanism, administered dose

## Abstract

**Objective:**

The present review was developed to critically evaluate the neuroprotective effects of edaravone for experimental rat models of spinal cord injury (SCI) and generalize the possible mechanisms.

**Methods:**

Systematic searches were carried out on databases including PubMed, Embase, Web of Science, Scopus, and Cochrane Library from their inception to March 2024. Controlled studies that assessed the neurological roles of edaravone on rats following SCI were selected. The Basso, Beattie, and Bresnahan (BBB) locomotor rating scale, residual white matter area, and malondialdehyde (MDA) level of the SCI rats were systematically searched by two reviewers.

**Results:**

Ten eligible publications were included. Meta-analyses showed increased BBB scores in edaravone-treated rats compared with control ones. The effect size gradually increased from day 7 (seven studies, *n* = 246, weighted mean difference (WMD) = 1.96, 95% confidence interval (CI) = 1.23 to 2.68, *P* < 0.00001) to day 28 (seven studies, *n* = 222, WMD = 4.41, 95% CI = 3.19 to 5.63, *P* < 0.00001) after injury and then maintained stably in the following time. Meanwhile, edaravone treatment was associated with an amendment in the spared area of white matter and a lowering in the MDA expression in the lesion area. The subgroup analyses revealed that rats treated with edaravone exhibited superior locomotor recovery in compression injury models than contusion ones. In network analyses, the surface under the cumulative ranking curve gradually increased up to a dose of 5–6 mg/(kg·d) of edaravone, after which it plateaued. Mechanism analysis suggested edaravone can ameliorate oxidative stress, mitigate neuroinflammation, and counteract neuron apoptosis and ferroptosis via multiple signaling pathways to exert its neuroprotective effects.

**Conclusion:**

Collectively, with a protective effect and a systematic action mechanism, edaravone warrants further investigation in SCI research and treatment. Nonetheless, in light of the limitations in the included studies, the findings in this review should be interpreted with caution.

**Systematic Review Registration:**

https://www.crd.york.ac.uk/PROSPERO/view/CRD42022374914.

## Introduction

Spinal cord injury (SCI) is a devastating event characterized by a high disability rate (Safdarian et al., 2023). Globally, approximately 20 million individuals are affected by varying degrees of SCI, with approximately 900,000 new cases of SCI occurring annually (Safdarian et al., 2023; James et al., 2019; Guan et al., 2023). Although its incidence is relatively low compared to other types of injury or degenerative disease, the adverse impact on individuals and the consequent societal and familial burden is immeasurable (Safdarian et al., 2023; James et al., 2019). Currently, the standard clinical treatment for SCI primarily comprises surgical interventions, pharmacological treatments (including neuroprotective agents, steroids, vasopressors, and anticoagulation prophylaxis), and rehabilitation therapies. However, the therapeutic benefits of these approaches appear to be limited, and recent evidence has increasingly questioned the efficacy of steroid use in SCI treatment (Jazayeri et al., 2022; Fehlings et al., 2022; Baroudi et al., 2024; Picetti et al., 2022). Consequently, there is an urgent need to develop novel and more effective therapeutic strategies for SCI.

Under physiological conditions, the endogenous antioxidant enzymes effectively counteract the limited number of oxidants primarily derived from cellular energy metabolism (Sies and Jones, 2020; Zaric et al., 2023). However, following SCI, this dynamic balance is broken, with serious, irreversible consequences. The disorders in microcirculatory and organelle function will manifest within a few hours after the initial injury, leading to the accumulation and large generation of reactive oxygen species (ROS) and, ultimately, oxidative stress (Tran et al., 2018; Zrzavy et al., 2021). Subsequently, oxidative stress can persist for an extended period and extensively participate in the pathological processes of secondary injury, initiating a sequential cascade of damage and disrupting the crucial microenvironment for tissue remodeling (Zrzavy et al., 2021; Peng et al., 2023). Therefore, the regulation of excessive oxidative stress always serves as a promising therapeutic strategy for the treatment of SCI.

Edaravone, a synthetic scavenger of ROS, is widely utilized in clinical settings for stroke management (Xu et al., 2019). Meanwhile, due to its proven safety and efficacy in treating amyotrophic lateral sclerosis, this therapeutic intervention has obtained regulatory approval for addressing this challenging disease in both Japan and the United States (Genge et al., 2022). Considering the pivotal role of oxidative stress in SCI, the application scope of edaravone in this field is also being explored. Several pre-clinical studies have investigated the efficacy of edaravone in treating this disease (Ishii et al., 2018; Pang et al., 2022). However, there is currently a paucity of integrated evidence specifically addressing the neuroprotective effects of edaravone for SCI. Furthermore, the mechanism responsible for the neuroprotective role of edaravone remains obscure. In this study, we conducted a comprehensive review to assess the neuroprotective role of edaravone in SCI and summarize the possible molecular mechanisms.

## Materials and methods

This systematic review was registered on PROSPERO (registration number CRD42022374914). Full details of the rationale and methods of this study were published elsewhere (Wang et al., 2023).

### Literature search

PubMed, Embase, Web of Science, Scopus, and Cochrane Library were systematically searched from their inception to March 2024. The reference lists of the selected studies and reviews were additionally scrutinized to encompass any other pertinent studies. According to the search strategy guidelines (Leenaars et al., 2012), “edaravone,” “MCI 186,” “spinal cord injuries,” “spinal cord injury,” “spinal cord compression,” “spinal cord trauma,” and “rat” were employed as a key term for retrieval (Sng et al., 2022; Zhou et al., 2022). The database search query is provided in Appendix 1 in supplemental material.

### Selection of studies

The initial database search results were imported into NoteExpress 3.9 software for filtering. Following the prespecified eligibility criteria, two independent evaluators conducted a preliminary screening of the literature based on titles and abstracts, followed by a comprehensive assessment of the full text of potentially eligible studies to determine their inclusion. The senior author made the final decision in the case of any disagreement between the two researchers (Yao et al., 2015).

### Eligibility criteria

#### Inclusion criteria

The inclusion criteria include the following: (1) controlled studies that assessed the neurological effects of edaravone on rat models of SCI without any restrictions on language, publication date, or publication status; (2) laboratory rats of any age, sex, or strain that underwent SCI induced by compression or contusion injury; (3) the experimental group was treated by edaravone with no restriction on dosage, formulation, and administration methods; (4) the control group received either vehicle, saline, saline with diluted dimethyl sulfoxide, or no treatment; and (5) reporting one of the following outcomes: Basso, Beattie, and Bresnahan (BBB) locomotor rating scale, spared white matter area, or malondialdehyde (MDA) (Sng et al., 2022).

#### Exclusion criteria

The following studies were excluded: (1) case reports, *in vitro* studies, clinical studies, reviews, and commentaries; (2) studies that used a rat model of SCI induced by non-traumatic ischemia, transection, laceration, traumatic root avulsion, or genetic modification; (3) studies combining edaravone with another treatment, without including a group treated with edaravone alone; (4) absence of control group; and (5) outcome assessments did not include BBB score, spared white matter area, or MDA.

#### Data extraction

Data extraction was independently conducted by two reviewers. Data collection includes the name of the first author, publication year, details of the study population, injury type and level, specifics of the intervention, and measurements. The proposed mechanisms and changes in related molecules were extracted to investigate the neuroprotective mechanism of edaravone. In studies encompassing multiple intervention groups, data from only the edaravone and negative control groups were extracted for analyses. The mean, standard deviations (SD), and sample sizes were collected from each group of animals. If the study data were represented visually without numerical values, we utilized GetData Graph Digitizer 2.26 (http://getdata-graph-digitizer.com) to extract the relevant statistical data from the graphical representations. In the event of missing data, the authors were contacted on a weekly basis. If no response was received from the original authors within 2 weeks, the related study was included but limited to qualitative analyses only (Yao et al., 2015).

#### Risk of bias assessment

The methodological quality of the included studies was assessed using SYRCLE’s RoB tool, a validated instrument for evaluating the risk of bias in laboratory animal experimentation within systematic reviews (Hooijmans et al., 2014). The checklist evaluates 10 items relating to selectivity bias, implementation bias, measurement bias, missed visit bias, reporting bias, and other biases. In each study, these domains were judged as “yes,” “no,” or “unclear,” indicating a low, high, or unclear risk of bias, respectively. The methodological quality assessment was conducted independently by two investigators, and any disagreements were resolved through consensus or the involvement of a third investigator (Zhou et al., 2022).

#### Statistical analysis

The data from the included studies were analyzed using RevMan 5.3 software. In studies with multiple intervention groups, we combined these groups to conduct a single pairwise comparison (Higgins and Green, 2011). The mean, SD, and sample size of animals in each group were used for comparisons. The effects of the interventions in each study were summarized using weighted mean differences (WMDs) or standardized mean differences, along with 95% confidence intervals (CIs). Heterogeneity among the included studies was assessed using the χ2 test and the Cochrane I^2^ (Higgins and Green, 2011). The random-effect model was employed due to the expected heterogeneity resulting from the exploratory character of the animal studies. Statistical significance was defined as a P value less than 0.05. Linear graphs were constructed using GraphPad Prism software to illustrate the dynamic WMDs of BBB scores and highlight the variation trend in BBB scores of both groups over time.

Subgroup analyses were conducted to investigate the factors that modify BBB scores, including species, gender, injury type, route of administration, and times and administered dosage. Meanwhile, we conducted sensitivity analyses to evaluate the robustness of our findings and to examine the sources of heterogeneity by excluding studies that lacked outcome assessor blinding, small-sample studies, and individual studies.

In order to investigate the optimal dose, we employed a network meta-analysis approach based on the Bayesian method using Stata 12.0 (StataCorp LP, College Station, Texas, USA) to simultaneously compare the therapeutic effects of different treatment regimens by integrating both direct and indirect evidence. We calculated the probability of each treatment regimen occupying a specific rank and subsequently ranked those treatments based on their surface under the cumulative ranking curve (SUCRA) (Owen et al., 2020).

## Results

### Study selection

**FIGURE 1:**
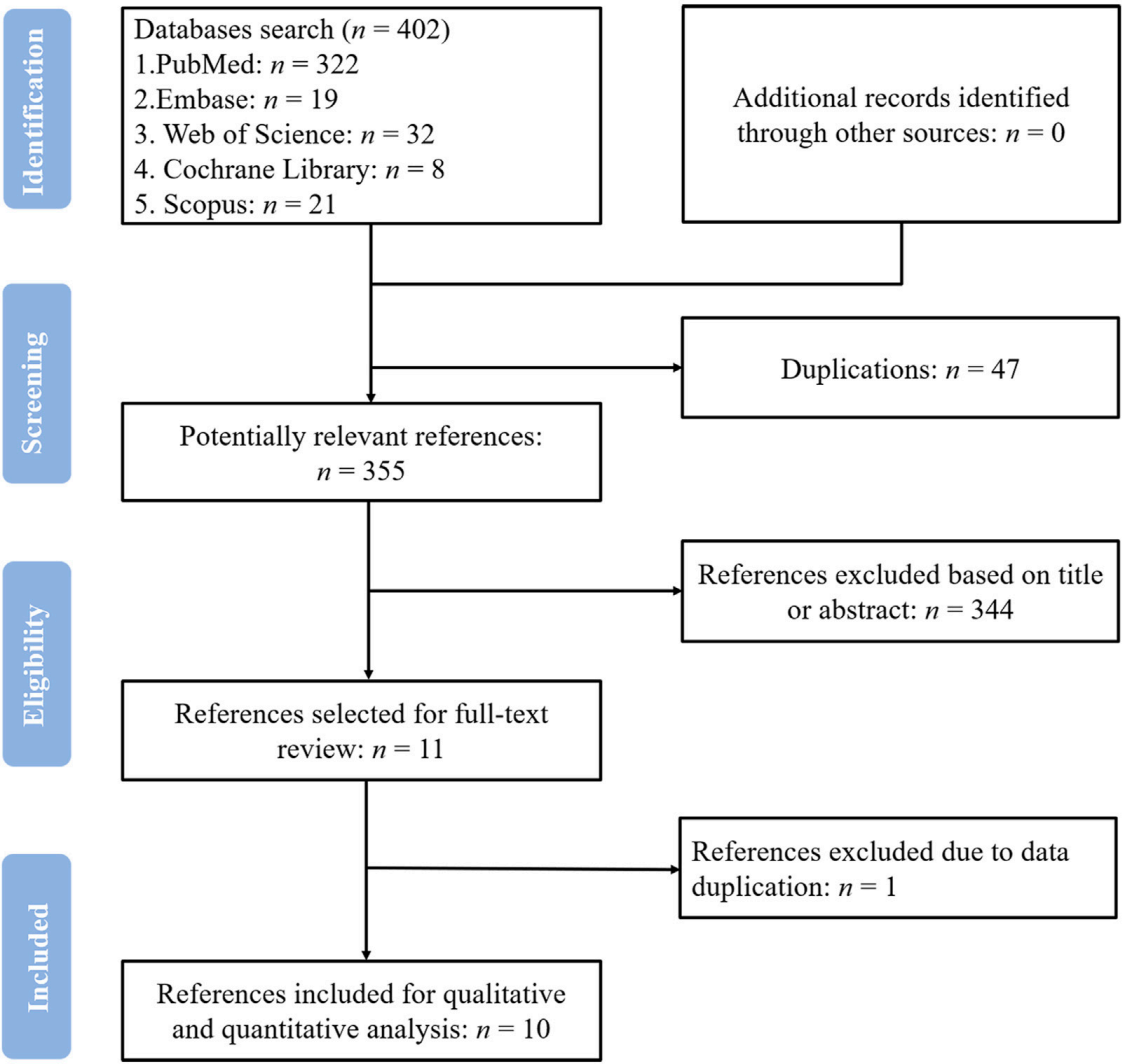
Summary of the literature identification and selection process.

The screening process for the study is shown in Figure 1. We identified 402 potentially relevant references after searching the databases described above. After eliminating duplicates and conducting an initial screening of the titles and abstracts, 11 publications were identified for full-text evaluation. Of them, one publication was excluded due to repetition of data (Zhao Z. J. et al., 2013). Ten publications (Ishii et al., 2018; Pang et al., 2022; Ohta et al., 2005; Ren et al., 2006; Ohta et al., 2011; Ozgiray et al., 2012; Wang et al., 2013; Song et al., 2015; Li et al., 2018; Xu et al., 2023), then, were eligible for this systematic review.

### Characteristics of the included studies

Of the 10 included publications, eight articles were published in English, and the remaining two were in Chinese (Ren et al., 2006; Li et al., 2018). The sample sizes varied between 24 and 191 rats. All studies except two (Pang et al., 2022; Song et al., 2015) used male rats. Eight publications used a weight-drop impactor to induce the SCI model, and the other two demonstrated an SCI model induced by compression of an aneurysm clip (Ishii et al., 2018; Xu et al., 2023). Almost all publications stated a trauma to the spinal cord at the T8–T11 segment. Six studies reported the source of edaravone procurement (Ishii et al., 2018; Pang et al., 2022; Ohta et al., 2005; Ren et al., 2006; Ohta et al., 2011; Xu et al., 2023). Placebo controls included normal saline, phosphate-buffered saline, and no treatment. The administered doses of edaravone ranged from 2 mg/(kg·d) to 75 mg/(kg·d). Four studies used intraperitoneal injection (Pang et al., 2022; Ohta et al., 2011; Li et al., 2018; Xu et al., 2023), five studies chose the intravenous route, with the remaining one not reporting this detail (Wang et al., 2013). Most studies administered edaravone within 2 h post-injury, although four studies included groups that received the initial edaravone injection at 4–24 h after trauma induction or prior to injury (Ishii et al., 2018; Wang et al., 2013; Song et al., 2015; Li et al., 2018). Four studies administered a single dose of edaravone (Ishii et al., 2018; Ohta et al., 2011; özgiray et al., 2012; Wang et al., 2013), while seven studies used repeated administration for 2–7 days after SCI (Pang et al., 2022; Ohta et al., 2005; Ren et al., 2006; Ohta et al., 2011; Song et al., 2015; Li et al., 2018; Xu et al., 2023). Additionally, two studies employed continuous infusion to administer edaravone (Table 1) (Ishii et al., 2018; Ohta et al., 2011).

**TABLE 1:** Characteristics of included studies.

Study	Animals	Injury model	Number of animals	Edaravone source	Groups	Administration time; treatment duration	Outcome
Xu B et al. (2023)	120 female SD rats (200–220 g)	T9 aneurysm clip compression 30 g × 30 s	40/40/40	Animal Center of the Chinese Academy of Sciences	A: Sham B: SCI C: SCI + edaravone (5 mg/kg, i.p.)	30 min after injury; once daily for 7 days	Behavioral: BBB scale, ICP, footprint analysis Histopathology: HE staining, LFB staining, immunofluorescence, Evans blue dye assays Other: Western blotting, serum biochemical assay, spinal cord tissue edema
Pang YL et al. (2022)	81 female Wistar rats (180 ± 20 g)	T9–T10 weight-drop impactor 10 g × 25 mm	27/27/27	Sigma	A: Sham B: SCI + vehicle C: SCI + edaravone (5 mg/kg, i.p.)	30 min after injury; once daily for 7 days	Behavioral: BBB scale, footprint analysis Histopathology: HE staining and immunofluorescence Other: Western blotting, cytokine array, and motor evoked potential
Li RB et al. (2018)	36 male SD rats (250–280 g)	T9–11 weight-drop impactor 12.5 mm	12/12/12	N/A	A: Sham + saline B: SCI + saline C: SCI + edaravone (5.42 mg/kg, i.p.)	Day 1 after injury; once daily for 7 days	Behavioral: BBB scale Histopathology: Nissl staining, immunohistochemistry Other: Western blotting
Ishii H et al. (2018)	104 male Wistar rats (370–420 g)	T10 compression 10 g × 10 min	7/7/7/7/7; 7/7/7/7/7; 7/7/7/7/7	Mitsubishi Chemical Group Corporation	1A: Sham 1B: SCI + saline 1C-1E: SCI + edaravone (3 mg/kg + 1 mg/kg/h, 3 mg/kg/h, 10 mg/kg/h, i.v.) 2A: Sham 2B: SCI + saline 2C-2E: SCI + edaravone (3 mg/kg + 3 mg/kg/h, i.v.) 3A: Sham 3B: SCI + saline 3C-3D: SCI + edaravone (3 mg/kg + 3 mg/kg/h, 10 mg/kg/h, i.v.)	1C-1E: administering 3 mg/kg edaravone 30 min prior to compression, followed by continuous infusion of 1 mg/kg, 3 mg/kg, or 10 mg/kg edaravone for 2 h 2C-2E: administering 3 mg/kg edaravone 30 min prior to compression, at the start of compression, and 10 min after decompression, respectively, followed by continuous infusion for 2 h 3C-3D: administering 3 mg/kg edaravone 30 min prior to compression, followed by continuous infusion of 3 mg/kg or 10 mg/kg edaravone for 2 h	Behavioral: BBB scale, inclined plane test Histopathology: immunohistochemistry Other: MDA, motor evoked potential
Song YY et al., 2015	80 female SD rats (250–300 g)	T9–T11 weight-drop impactor	20/20/20/20	N/A	A: SCI + PBS B: SCI + edaravone (3 mg/kg i.v.) C: SCI + neural stem cell D: SCI + edaravone + neural stem cell	6 h after injury; once daily for 7 days	Behavioral: BBB scale Other: fluorescent-gold retrograde tracing
Wang J et al., 2013	144 SD rats (200–250 g)	Weight-drop impactor	48/48/48	N/A	A: SCI + saline B: SCI + MP (30 mg/kg) C: SCI + edaravone (3 mg/kg)	2 h, 4 h, 6 h, 8 h, 10 h, 14 h, 16 h, and 24 h after injury; once	Behavioral: BBB scale Histopathology: HE staining, immunohistochemistry
Özgiray et al. (2012)	28 male Wistar rats (200–250 g)	T8–T10 weight-drop impactor 10 g × 50 mm	7/7/7/7	N/A	A: Sham B: SCI C: SCI + edaravone (3 mg/kg, i.p.) D: SCI + MP	1 h and 24 h after injury	Behavioral: neurological assessments Other: SOD, MDA, NO, GSH-Px, TAC
Ohta S et al. (2011)	191 male SD rats (295–325 g)	T9–10 weight-drop impactor 10 g × 6.25 mm or 12.5 mm	10/9/10/10/10/10; 20/20/15/15/15; 10/9/9/9/10	Mitsubishi Chemical Group Corporation	1A: SCI + saline 1B-1F: SCI + edaravone (1 mg/kg, 3 mg/kg, 5 mg/kg, 10 mg/kg, 20 mg/kg, i.v.) 2A: SCI + saline 2B: SCI + edaravone (3 mg/kg, i.v.) 2C-2E: SCI + edaravone (3 mg/kg + 1.5 mg/kg/h, 2.4 mg/kg/h, 3.0 mg/kg/h, i.v.) 3A: SCI + saline 3B: SCI + edaravone (3 mg/kg, i.v.) 3C: SCI + edaravone (3 mg/kg, i.v.) 3D and 3E: SCI + edaravone (3 mg/kg + 3.0 mg/kg/h, i.v.)	1B-1F: 5 min after injury; twice daily for 3 days 2B: 5 min after injury; once 2C-2E: administering 3 mg/kg edaravone 5 min after injury, followed by continuous infusion until sacrifice 3B: 5 min after injury; once 3C: 5 min after injury; twice daily for 3 days 3D and 3E: administering 3 mg/kg edaravone 5 min after injury, followed by continuous infusion for 24 h and 72 h, respectively	Behavioral: BBB scale Histopathology: Cresyl violet staining, LFB staining Other: MDA, plasma concentrations of edaravone
Ren XS et al. (2006)	30 male SD rats (250–300 g)	T9–T11 weight-drop impactor 10 g × 25 mm	15/15	Simcere	A: SCI + saline 1A: SCI + edaravone (5 mg/kg i.v.)	5 min after injury; once daily for 2 days	Behavioral: BBB scale, ICP Histopathology: LFB staining Other: MDA
Ohta S et al. (2005)	24 adult male SD rats (295–325 g)	T9–T10 weight-drop impactor 10 g × 25 mm	5/10/9	Mitsubishi Chemical Group Corporation	A: Sham B: SCI C: SCI + edaravone (5 mg/kg, i.v.)	5 min after injury; once daily for 2 days	Behavioral: BBB scale Histopathology: LFB staining Other: MDA

BBB, Basso, Beattie, and Bresnahan; DMSO, dimethyl sulfoxide; GSH-Px, glutathione peroxidase; HE, hematoxylin–eosin; IF, immunofluorescence; ICP, inclined plane test; i.p., intraperitoneal injection; i.v., intravenous injection; LFB, Luxol fast blue; MDA, malondialdehyde; MP, methylprednisolone; NO, nitric oxide; PBS, phosphate-buffered saline; SD, Sprague–Dawley; SCI, spinal cord injury; SOD, superoxide dismutase; TAC, total antioxidant capacity.

### Risk of bias within studies

As presented in Table 2, the included studies adequately addressed the items of “baseline characteristics” and “selective outcome reporting.” The items of “outcome assessor blinding” and “incomplete outcome data” were adequately addressed in 60% and 50% of the included studies, respectively. None or few studies reported on the items of “sequence generation,” “allocation concealment,” “investigator blinding,” “random housing,” and “random outcome assessment.” Additionally, no additional sources of bias were identified in the included studies.

**TABLE 2:** Risk of bias summary.

Study	1	2	3	4	5	6	7	8	9	10
Xu B et al. (2023)	Unclear	Yes	Unclear	Unclear	Unclear	Yes	Yes	Unclear	Yes	Yes
Pang YL et al. (2022)	Unclear	Yes	Unclear	Unclear	Unclear	Unclear	Yes	Unclear	Yes	Yes
Li RB et al. (2018)	Unclear	Yes	Unclear	Unclear	Unclear	Unclear	Unclear	Yes	Yes	Yes
Ishii H et al. (2018)	Unclear	Yes	Unclear	Unclear	Unclear	Unclear	Unclear	Yes	Yes	Yes
Song YY et al., 2015	Unclear	Yes	Unclear	Unclear	Unclear	Unclear	Yes	Unclear	Yes	Yes
Wang J et al., 2013	Unclear	Yes	Unclear	Unclear	Unclear	Unclear	Unclear	Yes	Yes	Yes
Özgiray et al. (2012)	Unclear	Yes	Unclear	Unclear	Unclear	Unclear	Unclear	Unclear	Yes	Yes
Ohta S et al. (2011)	Unclear	Yes	Unclear	Unclear	Unclear	Unclear	Yes	Yes	Yes	Yes
Ren XS et al. (2006)	Unclear	Yes	Unclear	Unclear	Unclear	Unclear	Yes	Yes	Yes	Yes
Ohta S et al. (2005)	Unclear	Yes	Unclear	Unclear	Unclear	Unclear	Yes	Unclear	Yes	Yes

“Yes” indicates a low risk of bias; “No” indicates a high risk of bias; “Unclear” represents an unclear risk of bias. This tool includes 10 questions: (1) sequence generation, (2) baseline characteristics, (3) allocation concealment, (4) random housing, (5) investigator blinding, (6) random outcome assessment, (7) outcome assessor blinding, (8) incomplete outcome data, (9) selective outcome reporting, (10) other sources of bias.

### Overall analysis of the effects of edaravone

#### Effects of edaravone on neurological recovery

**FIGURE 2:**
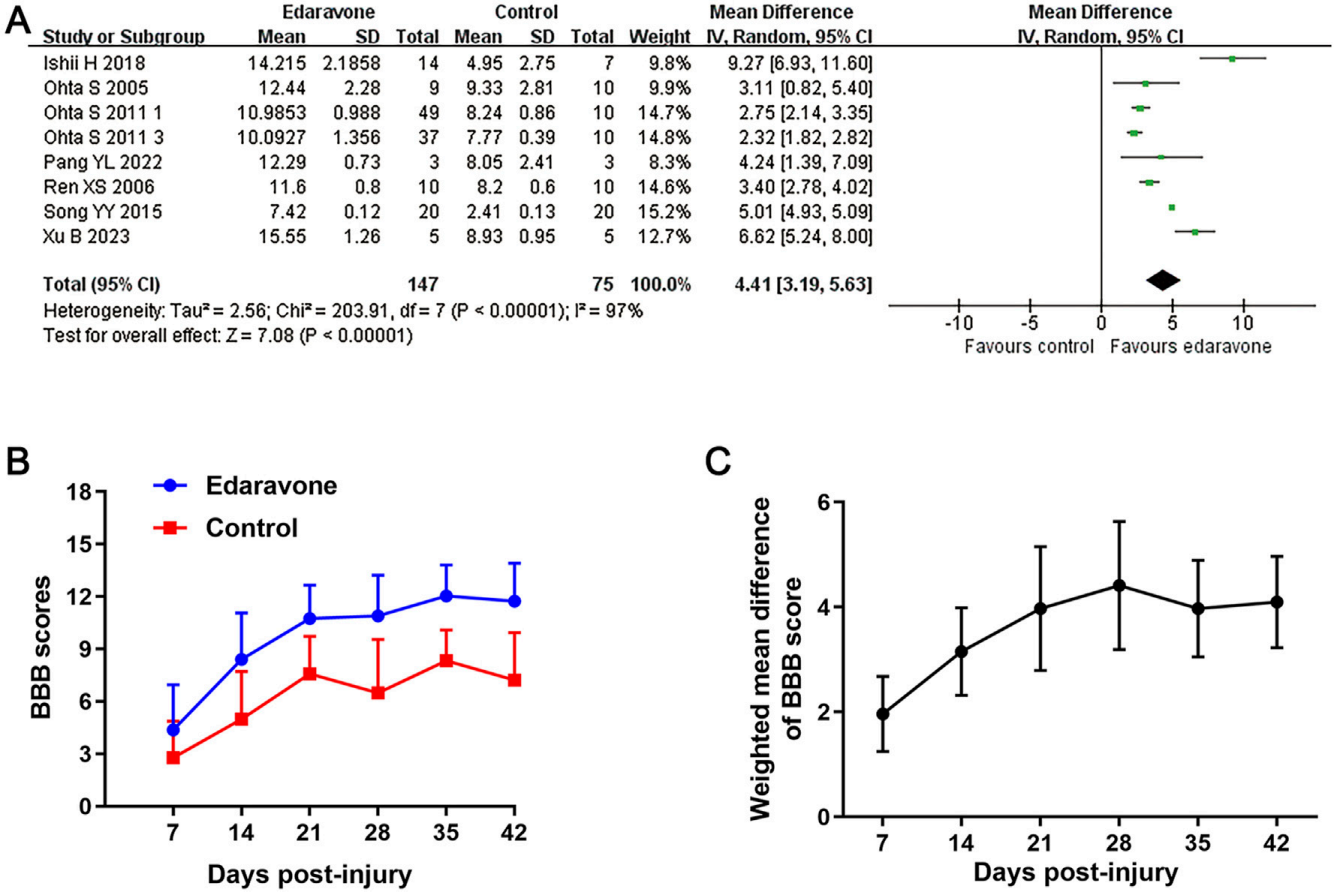
Overall analyses of the effects of edaravone on dynamic changes in neurological function. **(A)** Meta-analysis of BBB score at days 28 after SCI. **(B)** The BBB scores in each group over time. **(C)** The WMDs of BBB score between edaravone and control groups from day 7 to day 28 after SCI.

All studies, except one (özgiray et al., 2012), used BBB scores to evaluate locomotor recovery in rats following treatment with edaravone. The meta-analysis of BBB scores and the variation trend of BBB scores in both groups demonstrated that the edaravone-treated rats showed improved BBB scores compared to the control group. The WMDs between the two groups gradually increased from day 7 (seven studies, *n* = 246, WMD = 1.96, 95% CI = 1.23 to 2.68, *P* < 0.00001) to day 28 (seven studies, *n* = 222, WMD = 4.41, 95% CI = 3.19 to 5.63, *P* < 0.00001) after injury, and then maintained stably in the remaining time points (Figures 2A–C; Table 3).

**TABLE 3:** Summary of overall analyses of the effects of edaravone.

Outcome title	No. Of studies	No. Of animals	Weighted mean difference		Heterogeneity	
			95% CI	*P* value	*I* ^2^	*P* value
1 BBB scores	8	246				
1.1 BBB scale at day 7	8	246	1.96 [1.23, 2.68]	<0.00001	95	<0.00001
1.2 BBB scale at day 14	7	222	3.15 [2.31, 3.99]	<0.00001	93	<0.00001
1.3 BBB scale at day 21	6	182	3.97 [2.80, 5.15]	<0.00001	92	<0.00001
1.4 BBB scale at day 28	7	222	4.41 [3.19, 5.63]	<0.00001	97	<0.00001
1.5 BBB scale at day 35	5	172	3.97 [3.05, 4.89]	<0.00001	86	<0.00001
1.6 BBB scale at day 42	6	212	4.10 [3.23, 4.97]	<0.00001	91	<0.00001
2 Spared white matter	4	128	10.69 [6.24, 15.13]	<0.00001	94	<0.00001
3 MDA	5	85	−1.78 [−2.87, −0.68]	0.001	68	0.01

#### Effects of edaravone on tissue sparing

**FIGURE 3:**
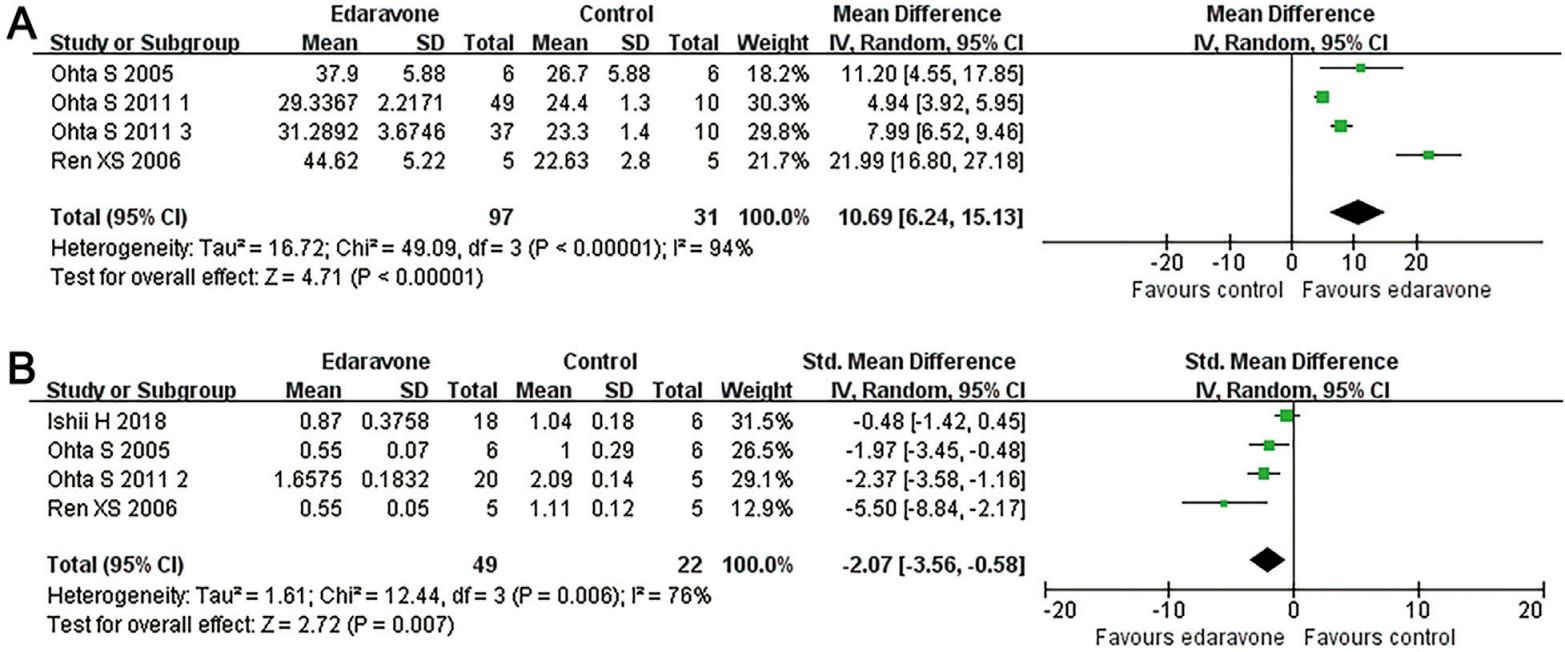
Overall analyses of the effects of edaravone on tissue sparing and MDA in the lesion area. **(A, B)** Meta-analysis of spared white matter area ratio and MDA.

Three studies reported the spared white matter areas in the injury epicenter using the Luxol fast blue method (Ohta et al., 2005; Ren et al., 2006; Ohta et al., 2011). All of them presented this outcome as the ratio of spared white matter area to total spinal cord area. Pooled results from those studies indicated an improvement in the spared white matter area in rats after edaravone treatment (three studies, n = 128, WMD = 10.69, 95% CI = 6.24 to 15.13, P < 0.00001; Figure 3A; Table 3).

#### Effects of edaravone on MDA level

Four studies reported the MDA content in the spinal cord tissue (Ishii et al., 2018; Ohta et al., 2005; Ren et al., 2006; özgiray et al., 2012). The pooled results demonstrated that edaravone intervention reduced MDA levels in the spinal cord compared to the placebo group (four studies, n = 71, WMD = −2.07, 95% CI = −3.56 to −0.58. P = 0.007; Figure 3B; Table 3).

#### Subgroup analyses of the effects of edaravone

**FIGURE 4:**
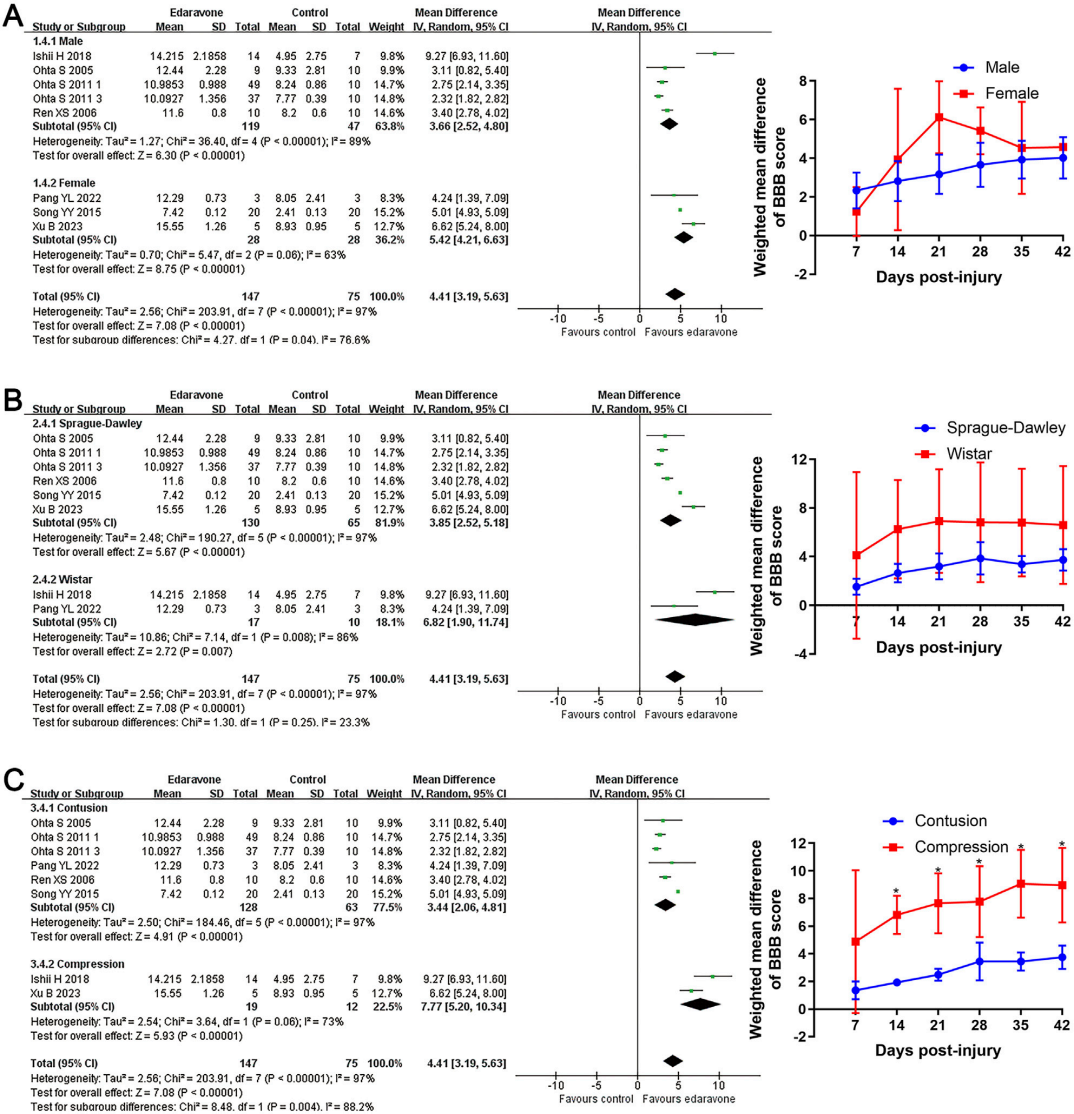
Subgroup analysis of BBB scores with respect to rat gender, rat strain, and injury model. **(A–C)** Subgroup analysis concerning rat gender, rat strain, and injury type at day 28 after SCI and the WMDs of BBB score in each subgroup over time. *p < 0.05.

**FIGURE 5:**
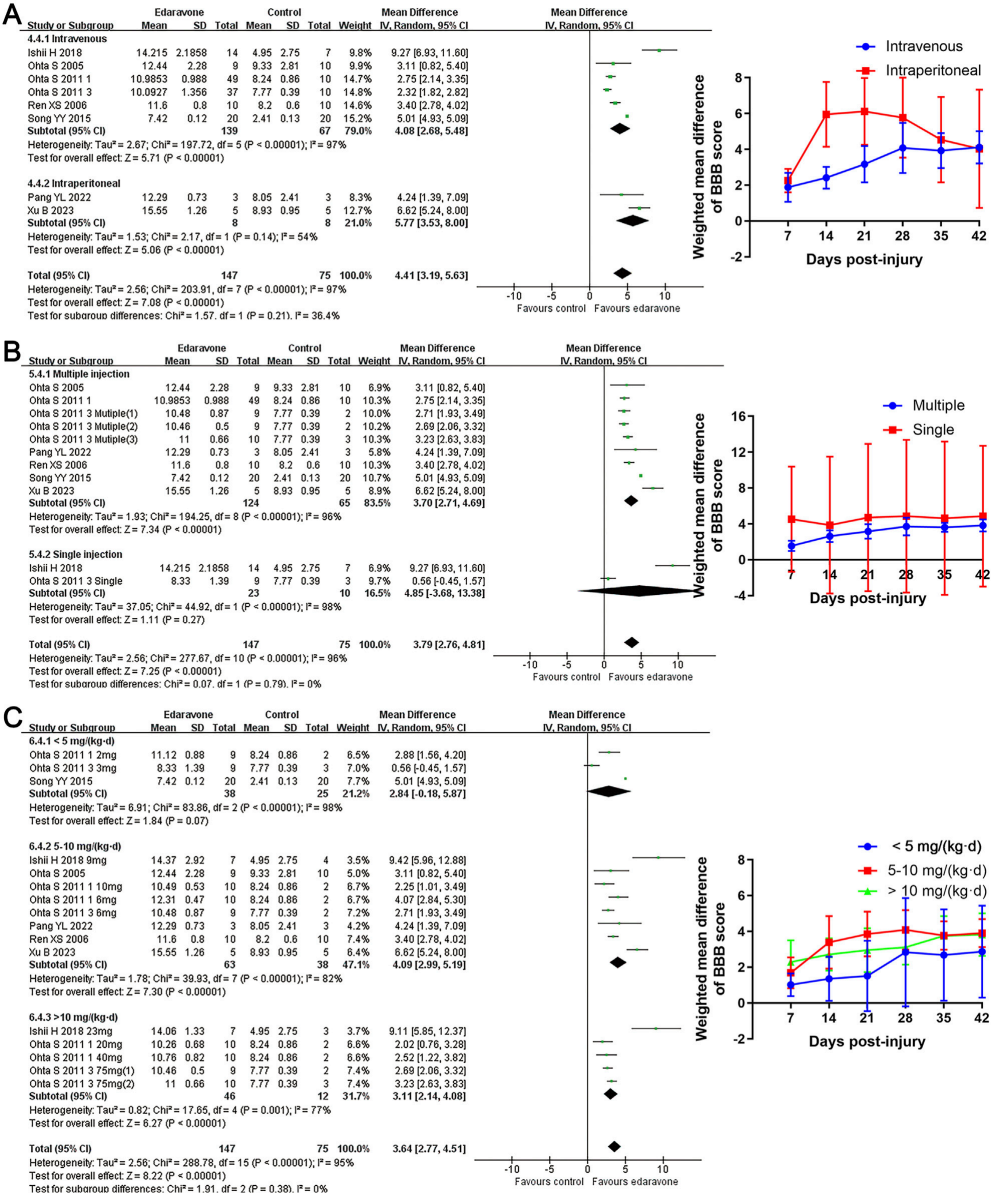
Subgroup analysis of BBB scores with respect to administration details. **(A–C)** Subgroup analysis concerning administration methods, administration times, and administered dose at day 28 after SCI and the WMDs of BBB score in each subgroup over time.

Subgroup analyses revealed no significant differences in locomotor recovery between rats based on gender, strain, number of injections (single versus multiple), or injection routes (intravenous versus intraperitoneal); however, locomotor recovery may be influenced by the type of injury (Figures 4, 5; Supplementary Table S1). Dose was argued to critically impact the efficacy of the pharmacological intervention. While our analysis suggested that edaravone at doses of 5–10 mg/(kg·d) only exhibited a weak tendency to improve BBB scores compared with doses of <5 mg/(kg·d), there was no statistically significant difference. Additionally, administering edaravone at doses exceeding 10 mg/(kg·d) did not result in any further improvement in BBB scores (Figure 5C; Supplementary Table S1). The relatively insufficient discrimination in doses may potentially impact the results of subgroup analyses; further analyses are required to explore the appropriate dose.

#### Network analysis of the effects of edaravone with different doses

**FIGURE 6:**
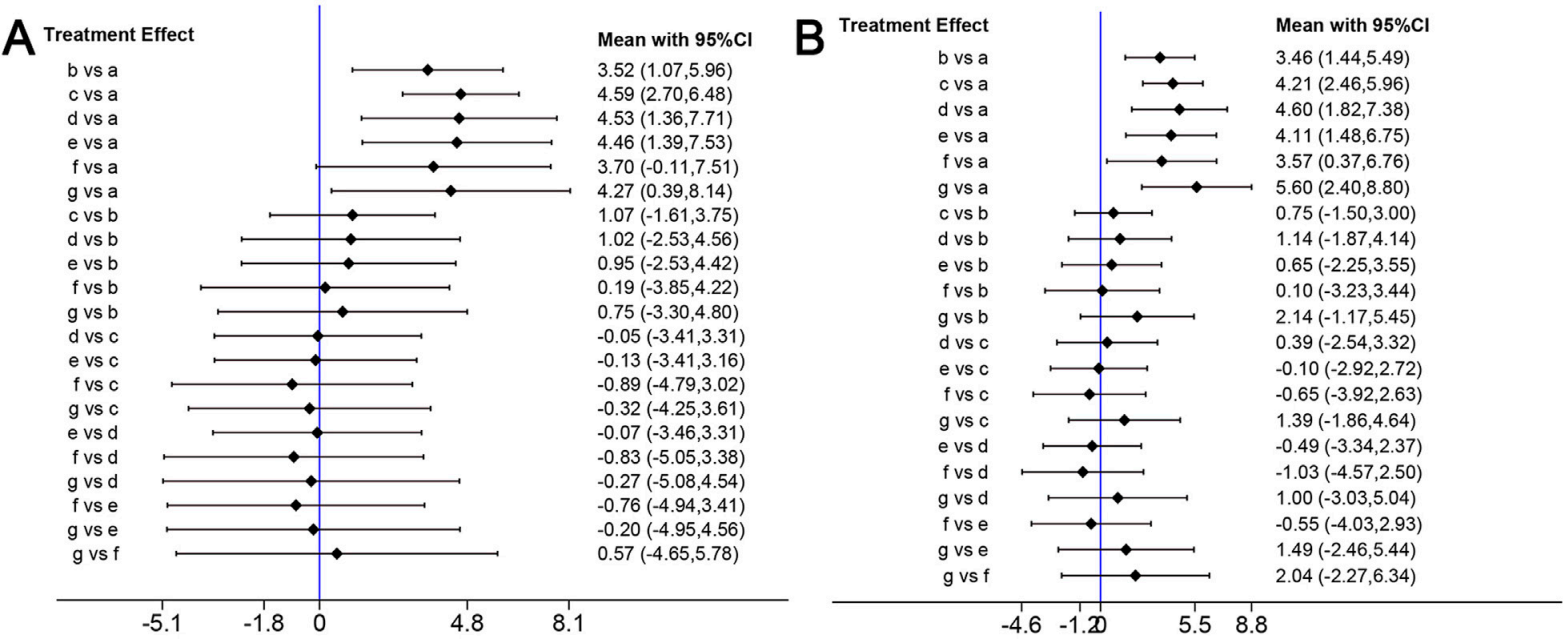
Network analysis of the effects of edaravone at different doses. **(A)** Forest plot of effect size in different edaravone doses according to data at day 28. **(B)** Forest plot of effect size in different edaravone doses according to data at day 42. a, control; b, 2–3 mg/(kg·d) edaravone; c, 5–6 mg/(kg·d) edaravone; d, 9–10 mg/(kg·d) edaravone; e, 20–23 mg/(kg·d) edaravone; f, 40 mg/(kg·d) edaravone; g, 75 mg/(kg·d) edaravone.

**FIGURE 7:**
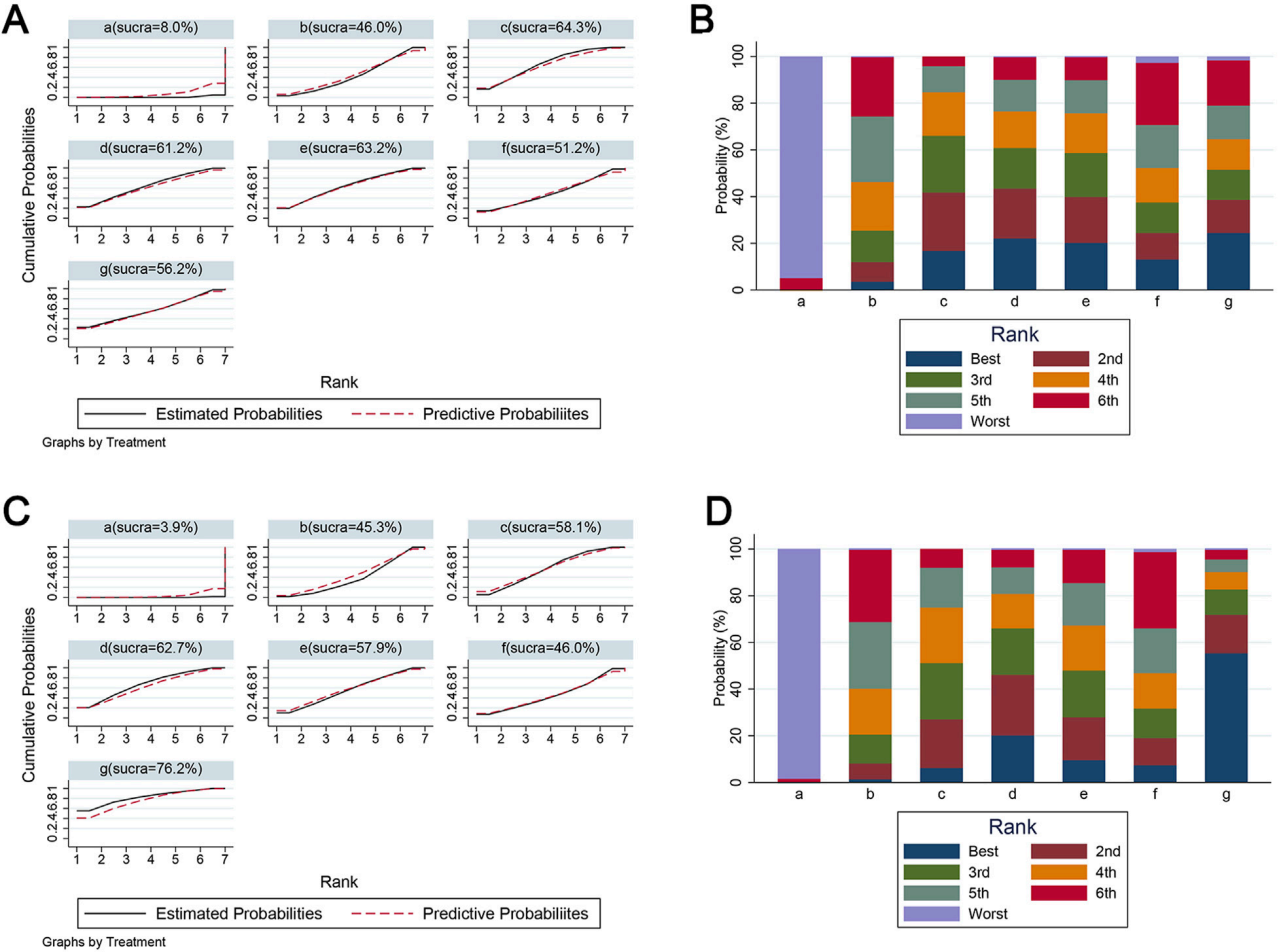
The SUCRA value and ranking probabilities of each treatment dose. **(A)** SUCRA value ranking of different administered doses according to data at day 28. **(B)** Histogram of the ranking probability of each treatment dose according to data at day 28. **(C)** SUCRA value ranking of different administered doses according to data at day 42. **(D)** Histogram of the ranking probability of each treatment dose according to data at day 42. a, control; b, 2–3 mg/(kg·d) edaravone; c, 5–6 mg/(kg·d) edaravone; d, 9–10 mg/(kg·d) edaravone; e, 20–23 mg/(kg·d) edaravone; f, 40 mg/(kg·d) edaravone; g, 75 mg/(kg·d) edaravone.

Because BBB scores trended stably from the fourth week after SCI, we conducted network analysis using BBB score data from day 28 to day 42 post-injury to compare the effects of edaravone at different doses (Zhou et al., 2019). At day 28 after injury, the forest plot indicated 5–6 mg/(kg·d) edaravone increased BBB scores compared with controls, and increasing the dose beyond 5–6 mg/(kg·d) did not produce better results (Figure 6A). The SUCRA values gradually increased as the daily dose increased initially, then trended stably from 5–6 mg/(kg·d) to the maximal dose (Figures 7A, B). The analysis of BBB scores on day 35 and day 42 after SCI yielded highly consistent results (Figure 6B, 7C; Supplementary Figure S1).

#### Sensitivity analysis

Sensitivity analyses were conducted by excluding studies that lacked outcome assessor blinding, small-sample studies, and individual studies. The heterogeneity of the inter-study statistics remained to be moderate or high after using all exclusion methods. The results of sensitivity analyses showed that the improvement of BBB scores after edaravone intervention remained largely unchanged across various exclusion strategies (Supplementary Table S2).

#### Therapeutic mechanisms

Table 4 shows the proposed mechanisms of edaravone in the included studies. Collectively, the neuroprotective mechanisms of edaravone for SCI primarily involve the mitigation of oxidative stress, suppression of neuroinflammation, reduction in neuronal loss, and attenuation of ferroptosis.

**TABLE 4:** The proposed mechanism of the protective effect of edaravone for SCI.

Study	Proposed mechanism	Effects
Xu B et al. (2023)	Autophagy, necroptosis, and blood–spinal cord barrier	1. Increased P62, decreased Beclin-1and LC3II/I 2. Decreased p-RIP1, p-RIP3, and p-MLKL 3. Increased ZO-1, β-catenin, and occludin
Pang YL et al. (2022)	Ferroptosis, neuroinflammation, and neuronal death	1. Increased GPX4 and xCT and decreased 5-LOX and ACSL4 2. Increased IL-10, IL-13, and adiponectin, and decreased Iba-1, and GFAP 3. Increased NeuN-positive cells
Li RB et al. (2018)	Collagen expression	Increased collagen type Ⅰ and Ⅳ
Ishii H et al. (2018)	Lipid peroxidation and neuronal death	1. Decreased MDA 2. Increased NeuN-positive cells
Song YY et al., 2015	Tissue repairing	Increased the survival and differentiation of transplanted NSCs
Wang J et al., 2013	Apoptosis	Increased Bcl-xL positive cells and decreased caspase-3 positive cells
Özgiray et al. (2012)	Oxidative stress	Increased SOD, GSH-Px, and TAC and decreased MDA and NO
Ohta S et al. (2011)	Inhibiting lipid peroxidation	Decreased MDA
Ren XS et al. (2006)	Inhibiting lipid peroxidation	Decreased MDA
Ohta S et al. (2005)	Inhibiting lipid peroxidation	Decreased MDA

ACSL4, Acyl-CoA, synthetase long-chain family member 4; Bcl-xl, B-cell lymphoma-extra large; GFAP, glial fibrillary acidic protein; GPX4, glutathione peroxidase 4; GSH-Px, glutathione peroxidase; MDA, malondialdehyde; NSC, neural stem cell; IL-10, interleukin-10; LC3, light chain 3; p-AKT, phosphorylated protein kinase B; p-ERK, phosphorylated extracellular signal-regulated kinase; p-MLKL, phosphorylated mixed-lineage kinase domain-like protein; p-RIP1, phosphorylated receptor-interacting protein 1; SOD, superoxide dismutase; TAC, total antioxidant capacity; xCT, system Xc-light chain; ZO-1, zonula occludens-1; 5-LOX, 5-lipoxygenase.

## Discussion

### Summary of evidence

The present systematic review included ten eligible studies to analyze the efficacy and potential mechanism of edaravone in treating SCI. The pairwise meta-analysis revealed a significant increase in BBB scores in rats treated with edaravone compared to the control group. The WMDs of BBB scores between the two groups exhibited a gradual increase from day 7 to day 28 post-injury, after which they stabilized during the subsequent observation period. Meanwhile, rats treated with edaravone showed a higher preservation rate of white matter areas at the lesion site. Additionally, the pooled data indicated that edaravone treatment resulted in a reduction in MDA levels in rats subjected to SCI. Those results demonstrated the positive efficacy of edaravone in enhancing locomotor recovery, reducing tissue damage, and alleviating oxidative stress in experimental SCI models.

The subgroup analyses indicated that the improvement in BBB scores was superior in compression injury models than in contusion models. Analyses with respect to the daily dose revealed no statistically significant differences in locomotor recovery across various daily doses of edaravone. However, the data suggested an initial upward trend followed by a plateau in the improvement of BBB scores as the dose increased. Based on this observation, we conducted a network meta-analysis to determine the optimal dosage of edaravone. The results confirmed the trends identified in the subgroup analysis and provided more comprehensive evidence to predict the appropriate dose of edaravone in treating experimental SCI.

### Edaravone dose

For many drugs, the augment of the therapeutic effect gradually slows with the increase of dosage, and the side effects significantly escalate as the dosage approaches its maximum levels (Riddle, 2000; Huscher et al., 2009). Administration of edaravone at doses of 1–75 mg/(kg·d) produced a protective effect for SCI models in included studies (Ishii et al., 2018; Ohta et al., 2011). Ohta et al. (2011) conducted a comparative study on the neuroprotective effect of different doses of edaravone in rats following SCI. Their findings demonstrated that the intravenous administration of 6 mg/kg edaravone daily resulted in significant improvements in locomotor function and tissue damage compared to control animals, while no further significant improvement was observed with the administration of 20 mg/(kg·d) and 40 mg/(kg·d) edaravone (Ohta et al., 2011). Consistently, our subgroup analysis suggested a tendency of improved BBB scores at doses of 5–10 mg/(kg·d) compared to the lower dose, but administration of the larger dose of edaravone did not result in further improvement in BBB scores. In network analyses, within daily doses of 5–6 mg/kg, the higher dose of edaravone is associated with a superior SUCRA, whereas administration of edaravone at doses of 9–10 mg/(kg·d), 20–23 mg/(kg·d), 40 mg/(kg·d), and 75 mg/(kg·d) did not further increase the SUCRA. Considering the potential side effects associated with high intervention doses, administration of 5–6 mg/(kg·d) edaravone may be considered appropriate for future pre-clinical investigations and could serve as a reference for potential clinical trials.

### Effects of edaravone on distinct injury models

Different injury mechanisms are associated with various pathological processes and may potentially impact the effectiveness of specific treatment modalities (Geremia et al., 2017). Our subgroup analyses revealed edaravone could result in a superior locomotory recovery in rats with compression injury, implying this agent is more appropriate for SCI induced by compression insult. Actually, this phenomenon is predictable due to the pathological features of compression injury. There are several similarities in the pathological alterations observed in compression and contusion SCIs. Prolonged compression, however, can lead to a reduction in blood flow, blood oxygen level, and glucose supply at the site of compression, thereby inducing electron leakage in mitochondria and the production of free radicals (Abraham et al., 1985; Ueta et al., 1992; Okon et al., 2013). Subsequently, the reperfusion event results in an enlargement of the damaged area and degree by boosting ROS production (Abraham et al., 1985). The processes of ischemia and reperfusion disrupt the metabolism of free radicals and energy at the injury site and powerfully contribute to excessive oxidative stress (Ling et al., 2021). Therefore, compression SCIs may exhibit more pronounced oxidative stress than contusion injuries. As mentioned above, edaravone is well-known for its excellent antioxidant effect. Then, the powerful antioxidant role endows edaravone to elicit superior applicability in compression SCIs. Nevertheless, this hypothesis needs further studies to verify.

### Potential therapeutic mechanisms

Based on the proposed mechanisms outlined in the included studies and relevant supporting evidence, the potential underlying mechanisms of edaravone are as follows:

#### Blunting oxidative stress

Following SCI, a substantial accumulation of free radicals occurs rapidly and persistently at the site of injury, triggering a cascade of pathological events during the secondary injury phase (Zrzavy et al., 2021; Peng et al., 2023; Yu et al., 2023). Superoxide dismutase (SOD), a type of metalloenzyme, plays a pivotal role in scavenging free radicals of superoxide anions and serves as a crucial component in the detoxification of ROS (Wang et al., 2018). While MDA is mainly produced as a product of the lipid peroxidation reaction triggered by ROS, it subsequently amplifies the occurrence of secondary harmful reactions and the generation of ROS (Wang et al., 2019). The antioxidative capacity of edaravone has been proved in numerous central nervous system (CNS) diseases (Jiao et al., 2015; Ahmadinejad et al., 2017; Jaiswal, 2019). Five of the included studies reported a decreased level of MDA or an enhanced SOD content in rats with SCI treated with edaravone (Ishii et al., 2018; Ohta et al., 2005; Ren et al., 2006; Ohta et al., 2011; özgiray et al., 2012). The meta-analysis revealed that edaravone resulted in a reduction in MDA levels in the spinal cord compared to the placebo group, verifying the favorable antioxidative role of edaravone in treating SCI.

The combination of a direct and indirect role in free radicals gives edaravone a powerful antioxidative property (Watanabe et al., 2018; Ismail et al., 2020). At a physiological pH, approximately half of edaravone exists as edaravone anions, allowing this agent to directly trap hydroxyl radicals and quench active oxygen by electron transfer or reaction with oxygen molecules (Watanabe et al., 2018; Higashi et al., 2006). In addition, various signaling pathways act as intermediary links for the antioxidant mechanism of edaravone (Ismail et al., 2020; Zhang et al., 2019). Nuclear factor erythroid 2-related factor 2 (Nrf2) and sirtuin 1 (SIRT1) can modulate mitochondrial homeostasis and antioxidant enzyme activity to defend against oxidative stress, while inactivation of these molecules occurs in SCI and related CNS diseases (Samarghandian et al., 2020; Jiang et al., 2023). In a pheochromocytoma cell 12 (PC12) model of neurotoxicity, edaravone was shown to enhance Nrf2 expression, reduce the generation of ROS, and promote cell survival, which was abolished by Nrf2 gene knockdown (Shou et al., 2019). Meanwhile, edaravone treatment has been shown to activate Nrf2, heme oxygenase 1, and SIRT1 in experimental models of traumatic brain injury, amyotrophic lateral sclerosis, and stroke (Ismail et al., 2020; Cui X. et al., 2022). Collectively, the unique structure of edaravone, along with the involvement of Nrf2 and SIRT1, potentially mediates its role in attenuating oxidative stress to mitigate disorders in neurological function and tissue structure in SCI.

#### Eliminating neuroinflammation

Microglia, the resident immune cells in the CNS, respond to the initial injury of the spinal cord within a few hours and facilitate the activation of astrocytes as well as infiltration of peripheral immune cells at the injury site. Subsequently, an inflammatory cascade is initiated (Hu et al., 2023). Scavenging of ROS is of significance for ameliorating neuroinflammatory responses (Zrzavy et al., 2021). Correspondingly, Pang et al. (2022) demonstrated that edaravone effectively suppresses the activation of microglia and astrocytes, thereby mitigating inflammatory responses in SCI. Moreover, the expression of anti-inflammatory factors, such as IL-10 and IL-13, was upregulated by edaravone to effectively attenuate neuroinflammation (Pang et al., 2022). Nuclear transcription factor-κB (NF-κB) and the NOD-like receptor family pyrin domain-containing 3 (NLRP3) inflammasome function as downstream signals of ROS, playing pivotal roles in regulating persistent inflammatory responses during SCI (Gao et al., 2019; Liu et al., 2020). In HT22 cells stimulated by amyloid β or hydrogen peroxide, edaravone was shown to ameliorate inflammatory response and cell apoptosis by diminishing ROS generation, inhibiting NF-κB p65 phosphorylation, and suppressing NLRP3 inflammasome activation (Zhao Z. Y. et al., 2013; Guo et al., 2023). Edaravone reduced the expression of NLRP3, caspase-1, and associated speck-like protein containing a caspase-recruitment domain to blunt the microglial activation induced by lipopolysaccharide (Li J. et al., 2021). Evidence from the *in vivo* studies confirmed the inhibitory effect of edaravone on ROS generation, NLRP3 inflammasome, and nuclear transcription of NF-κB in multiple neurological disorders (Akiyama et al., 2017; Miao et al., 2020; Meng et al., 2021). Therefore, the suppression of neuroinflammation by edaravone may be attributed to its ability to inhibit free radicals and downstream signaling pathways, including NF-κB and NLRP3 inflammasome.

#### Attenuating neuronal apoptosis

The generation of large numbers of free radicals and a vigorous inflammatory cascade response following injury can notably disturb the organelle function and homeostasis in neuronal cells, leading to numerous neuron apoptosis (Zrzavy et al., 2021; Sun et al., 2024). In the experimental treatment of SCI, it was reported that edaravone increased the survival rate of neuron cells in the lesion area (Ishii et al., 2018; Pang et al., 2022). Our meta-analysis also revealed edaravone can mitigate tissue damage in the lesion area. The B-cell lymphoma-extra-large (Bcl-XL) and caspases are involved in the cell apoptosis event resulting from oxidative stress (Liu et al., 2022). Administration of edaravone could result in a significant decrease in caspase-3 positive cells and a rise in Bcl-XL levels in rats with SCI (Wang et al., 2013). As mentioned above, Nrf2 and SIRT1 are important regulators of oxidative stress (Samarghandian et al., 2020; Jiang et al., 2023). Interestingly, edaravone was suggested to effectively boost the activation of Nrf2 and SIRT1 to improve the neuron apoptosis caused by excessive generation of ROS during brain injury (Shou et al., 2019; Li X. et al., 2021; Cui X. P. et al., 2022). Collectively, a rational molecular chain comprising Nrf2 (or SIRT1), ROS, and Bcl-XL (or caspase-3) may account for the anti-apoptotic mechanism of edaravone in treating SCI and related CNS diseases.

#### Repressing ferroptosis

The rapid and persistent oxidative stress can induce iron overload, impairing the activity of glutathione peroxidase 4 and reinforcing the buildups of ROS in return. This leads to structural damage to the cellular membrane and ultimately causes cell death (Feng et al., 2021; Zhang et al., 2022). Notably, edaravone treatment after SCI was suggested to upregulate anti-ferroptosis proteins, including glutathione peroxidase 4 and system Xc-light chain, and exert inhibitory effects on pro-ferroptosis proteins (Pang et al., 2022), implying that an anti-ferroptosis mechanism participates in the neuroprotective effect of edaravone. The detailed molecular mechanism behind the anti-ferroptosis of edaravone remains to be further illustrated in future studies.

**FIGURE 8:**
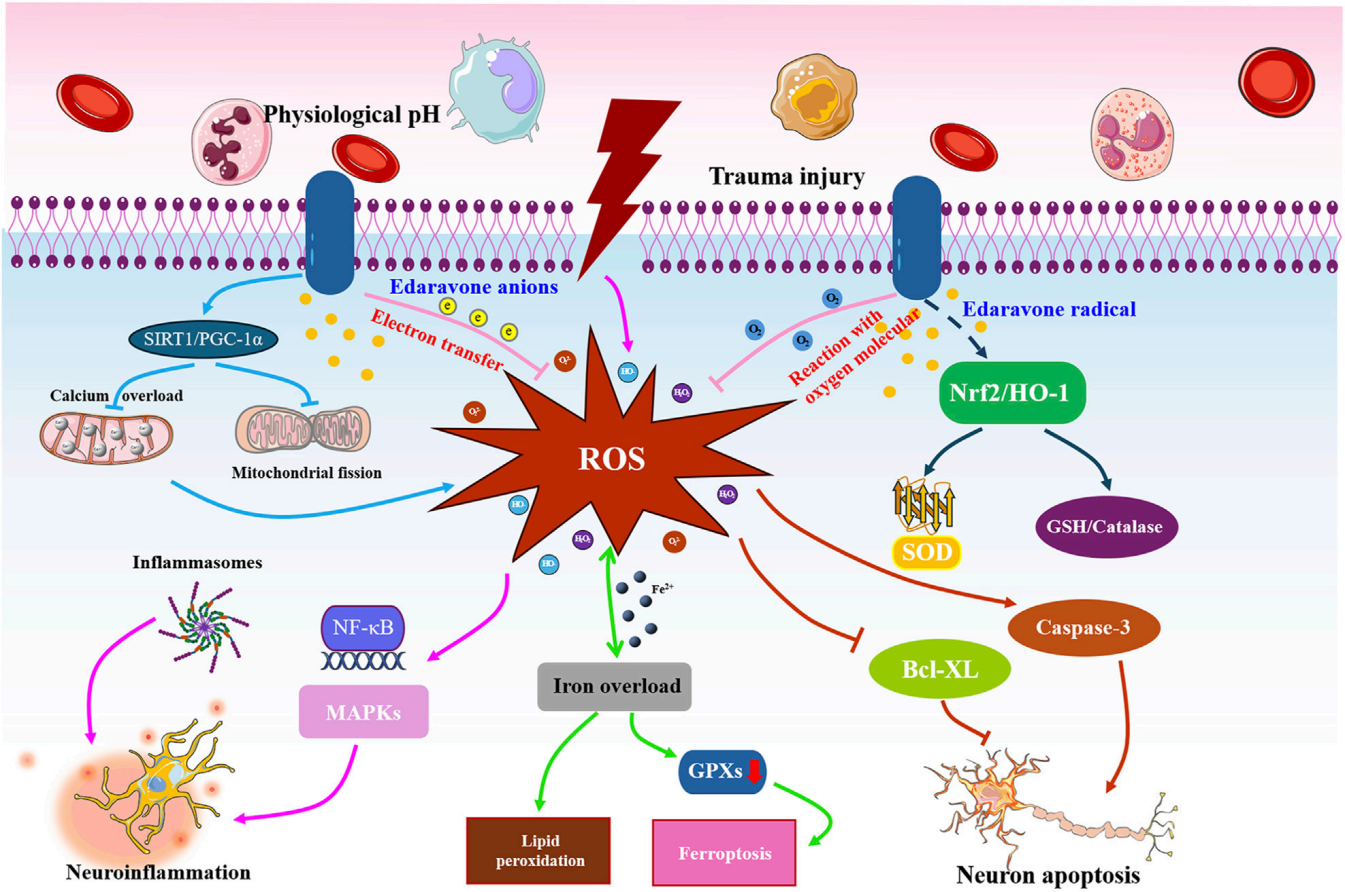
Potential action mechanisms of edaravone against SCI. Following the primary insult, large quantities of free radicals will rapidly and persistently accumulate at the site of injury, leading to neuroinflammation, neuronal apoptosis, and cell ferroptosis. Edaravone executes a satisfactory role in blunting oxidative stress by transferring electrons, reacting with oxygen molecules, and activating SIRT1 and Nrf2 pathways. Partly depending on the antioxidant effects, edaravone then inhibits the NLRP3 inflammasome or NF-κB signaling to counteract neuroinflammatory responses, regulate the caspase, Bax, and caspase family proteins to attenuate neuronal cell apoptosis, and modulate the ferroptosis-related proteins to suppress ferroptosis, finally exerting neuroprotective roles in treating SCI rats.

Taken together, we hypothesize that the core mechanism through which edaravone exerts its neuroprotective effects is by attenuating oxidative stress. Partly due to its antioxidant effects, edaravone then counteracts neuroinflammatory responses, attenuates neuronal cell apoptosis, and suppresses ferroptosis through diverse molecular pathways for the treatment of SCI (Figure 8). However, more high-quality studies are required to verify this hypothesis.

### Strengths and limitations

To the best of our knowledge, this meta-analysis represents the first quantitative analysis of edaravone’s therapeutic efficacy in treating SCI. In this review, our primary focus is on the dynamic fluctuations of BBB scores following intervention with edaravone, while also addressing its tissue-protective and antioxidant effects in the context of SCI. The network analyses provide a possible reference for the candidate dose of edaravone. Additionally, we have proposed a mechanistic framework elucidating the neuroprotective effects of edaravone in SCI through an amalgamation of systematic and traditional review approaches, culminating in the construction of a schematic representation depicting these underlying mechanisms. In order to conduct subsequent pre-clinical investigations and assess its potential application in clinical settings, it is essential to comprehend the effectiveness, specifics of administration, and mechanism of action for a particular agent. Our study conducted a comprehensive analysis of the parameters associated with edaravone, aiming to advance basic research on SCI and contribute to the discovery of novel clinical therapies for this condition.

Several limitations should be considered. First, due to the limited data available from studies investigating the efficacy of edaravone in SCI animal models, we selected the outcomes of BBB score, white matter integrity, and MDA for our protocol development and subsequent analyses. Combining these three indicators has certain advantages in demonstrating edaravone’s efficacy by providing complementary validation through objective behavioral scales, pathological assessments, and biochemical measurements. However, these indicators are still insufficient for a comprehensive evaluation of the efficacy of edaravone. Future research is needed to further enrich other dimensions of data and facilitate a more thorough understanding of the effects of edaravone on SCI. Second, we selected data with BBB scores from day 28–42 post-injury for network analysis because BBB scores tended to stabilize after day 28. This approach allows for a focused evaluation of the efficacy of different drug doses on functional recovery at these important timepoints. However, this method was insufficient in reflecting the differential effects of various doses on early and long-term functional recovery following SCI, potentially leading to an incomplete understanding of the efficacy of different doses. Third, although some factors were reported to modulate edaravone’s efficacy, subgroup analyses showed that most did not significantly impact the therapeutic effect of edaravone. It is important to note that subgroup analysis should be seen as a tool for generating hypotheses rather than confirming them. More studies aimed at examining differential effects across subgroups in more detail are needed. Fourth, similar to many other meta-analyses on animal models, our analyses reveal significant heterogeneity in treatment outcomes. Based on the results from subgroup analyses and sensitivity analyses, the high heterogeneity between studies appeared to be associated with the differences in injection dose, administration mode, injury type, rat gender and strain, and the methodological quality of studies. Given the substantial heterogeneity among the studies, the findings of this review should be interpreted with caution.

## Conclusion

In conclusion, pooled data analysis from this review supports using edaravone treatment to enhance motor recovery, ameliorate tissue damage, and reduce oxidative stress in rat models of SCI. The recommended dosage is 5–6 mg/kg daily. Based on the literature data, edaravone can alleviate oxidative stress, suppress neuroinflammation, reduce neuronal loss, and counteract ferroptosis in the treatment of SCI. Due to its protective effect and systematic action mechanism, edaravone shows promise as a neuroprotective agent for the research and treatment of SCI. However, considering the limitations in the design of the included studies, further high-quality research is needed to support the effectiveness of edaravone as a therapeutic intervention for SCI.

## Data Availability

The original contributions presented in the study are included in the article/Supplementary Material, further inquiries can be directed to the corresponding author.
