# Supplementary material for: Neuroprotective effect and possible mechanism of edaravone in rat models of spinal cord injury: a systematic review and network meta-analysis
Source: Front Pharmacol. 2025 Apr 7;16:1538879. doi: 10.3389/fphar.2025.1538879 (PMC12009846; doi:10.3389/fphar.2025.1538879)
Supplement: Supplementary file 1 [file DataSheet1.docx]

**Neuroprotective effect and possible mechanism of** **edaravone in rat models of spinal cord injury: a** **systematic review and network meta-analysis**

**SUPPLEMENTARY MATERIAL**


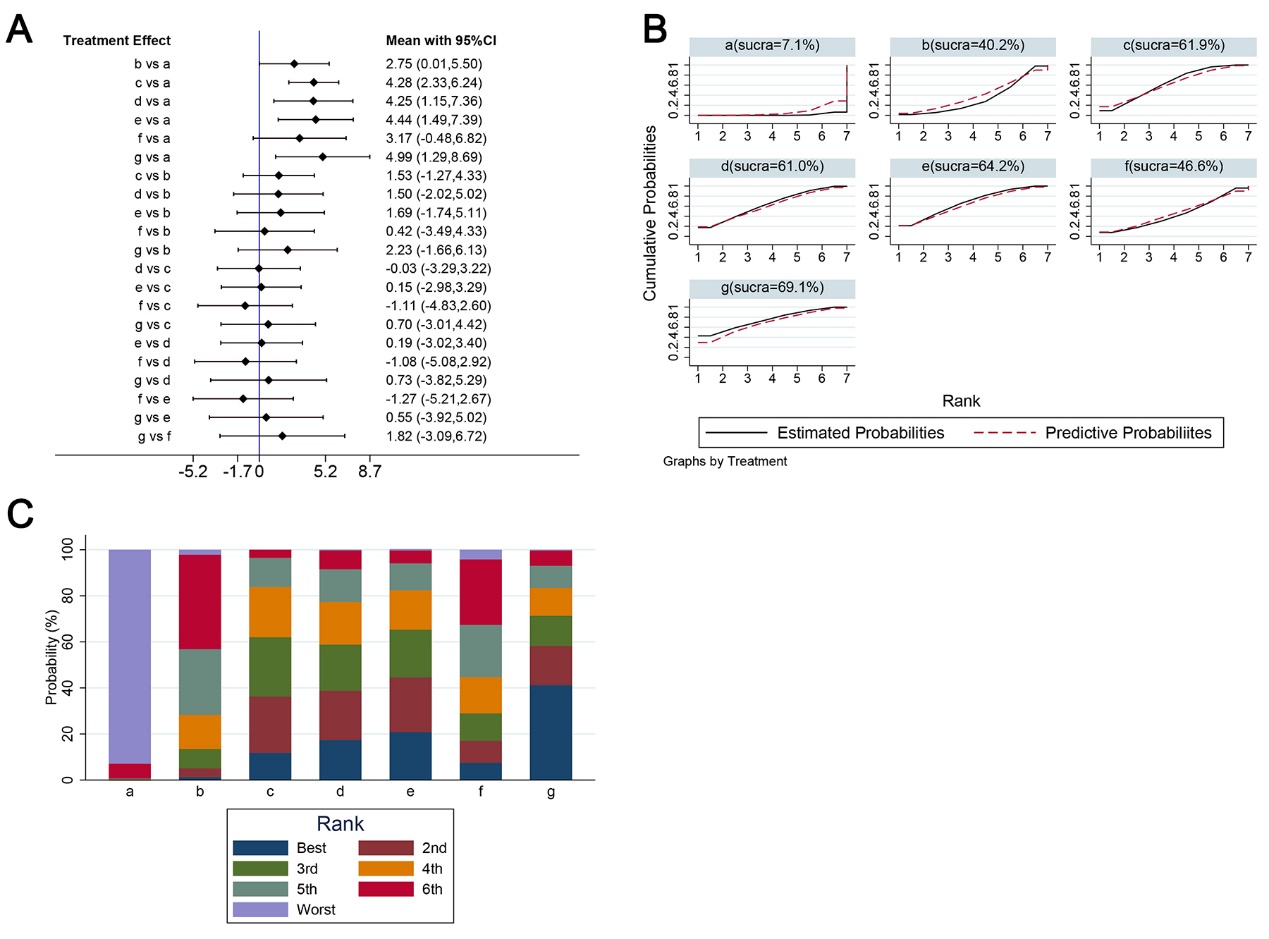


**Fig. S1 Network analysis of the effects of** **edaravone at different doses.** (A) Forest plot of effect size in different edaravone doses according to data at day 35. (B) SUCRA value ranking of different administration doses according to data at day 35. (C) Histogram of ranking probability of each treatment dose according to data at day 35.

**Table S1. Subgroup analyses of the effects of edaravone.**

| **Subgroup title** | **No. of studies** | **No. of animals** | **Weighted mean difference** | | **Heterogeneity** | | **Subgroup difference** |
| --- | --- | --- | --- | --- | --- | --- | --- |
|  |  |  | **95% CI** | ***P* value** | ***I*^2^** | ***P* value** |  |
| 1 Rat gender | 8 | 246 |  |  |  |  |  |
| 1.1 BBB scale at 7^th^ day | 8 | 246 |  |  |  |  | *P* = 0.18 |
| 1.1.1 Male | 5 | 190 | 2.33 [1.39, 3.26] | < 0.00001 | 93 | < 0.00001 |  |
| 1.1.2 Female | 3 | 56 | 1.24 [-0.03, 2.51] | 0.05 | 87 | 0.0006 |  |
| 1.2 BBB scale at 14^th^ day | 7 | 222 |  |  |  |  | *P* = 0.56 |
| 1.2.1 Male | 4 | 166 | 2.82 [1.79, 3.85] | < 0.00001 | 89 | < 0.00001 |  |
| 1.2.2 Female | 3 | 56 | 3.94 [0.27, 7.06] | 0.004 | 97 | < 0.00001 |  |
| 1.3 BBB scale at 21^st^ day | 6 | 182 |  |  |  |  | *P* = 0.007 |
| 1.3.1 Male | 4 | 166 | 3.17 [2.15, 4.19] | < 0.00001 | 89 | < 0.00001 |  |
| 1.3.2 Female | 2 | 16 | 6.12 [4.26, 7.99] | < 0.00001 | 38 | 0.20 |  |
| 1.4 BBB scale at 28^th^ day | 7 | 222 |  |  |  |  | *P* = 0.04 |
| 1.4.1 Male | 4 | 166 | 3.66 [2.52, 4.80] | < 0.00001 | 89 | < 0.00001 |  |
| 1.4.2 Female | 3 | 56 | 5.42 [4.21, 6.63] | < 0.00001 | 63 | 0.06 |  |
| 1.5 BBB scale at 35^th^ day | 5 | 172 |  |  |  |  | *P* = 0.64 |
| 1.5.1 Male | 4 | 166 | 3.93 [2.94, 4.91] | < 0.00001 | 88 | < 0.00001 |  |
| 1.5.2 Female | 1 | 6 | 4.54 [2.15, 6.93] | 0.0002 |  |  |  |
| 1.6 BBB scale at 42^nd^ day | 6 | 212 |  |  |  |  | *P* = 0.30 |
| 1.6.1 Male | 4 | 166 | 4.02 [2.95, 5.09] | < 0.00001 | 88 | < 0.00001 |  |
| 1.6.2 Female | 2 | 46 | 4.58 [4.51, 4.65] | < 0.00001 | 0 | 0.74 |  |
| 2 Rat strain | 8 | 246 |  |  |  |  |  |
| 2.1 BBB scale at 7^th^ day | 8 | 246 |  |  |  |  | *P* = 0.46 |
| 2.1.1 Sprague Dawley | 6 | 219 | 1.53 [0.88, 2.18] | < 0.00001 | 94 | < 0.00001 |  |
| 2.1.2 Wistar | 2 | 27 | 4.10 [-2.74, 10.94] | 0.24 | 96 | <0.00001 |  |
| 2.2 BBB scale at 14^th^ day | 7 | 222 |  |  |  |  | *P* = 0.09 |
| 2.2.1 Sprague Dawley | 5 | 195 | 2.64 [1.89, 3.40] | < 0.00001 | 93 | < 0.00001 |  |
| 2.2.2 Wistar | 2 | 27 | 6.25 [2.20, 10.30] | < 0.002 | 61 | 0.11 |  |
| 2.3 BBB scale at 21^st^ da | 6 | 182 |  |  |  |  | *P* = 0.10 |
| 2.3.1 Sprague Dawley | 4 | 155 | 3.18 [2.12, 4.24] | < 0.00001 | 91 | < 0.00001 |  |
| 2.3.2 Wistar | 2 | 27 | 6.91 [2.65, 11.17] | 0.001 | 81 | 0.02 |  |
| 2.4 BBB scale at 28^th^ day | 7 | 222 |  |  |  |  | *P* = 0.25 |
| 2.4.1 Sprague Dawley | 5 | 195 | 3.85 [2.52, 5.18] | < 0.00001 | 97 | < 0.00001 |  |
| 2.4.2 Wistar | 2 | 27 | 6.82 [1.90, 11.74] | 0.007 | 86 | 0.008 |  |
| 2.5 BBB scale at 35^th^ day | 5 | 172 |  |  |  |  | *P* = 0.13 |
| 2.5.1 Sprague Dawley | 3 | 145 | 3.37 [2.70, 4.05] | < 0.00001 | 78 | 0.003 |  |
| 2.5.2 Wistar | 2 | 27 | 6.79 [2.36, 11.22] | 0.003 | 85 | 0.01 |  |
| 2.6 BBB scale at 42^nd^ day | 6 | 212 |  |  |  |  | *P* = 0.25 |
| 2.6.1 Sprague Dawley | 4 | 185 | 3.73 [2.85, 4.61] | < 0.00001 | 93 | < 0.00001 |  |
| 2.6.2 Wistar | 2 | 27 | 6.59 [1.76, 11.43] | 0.007 | 81 | 0.02 |  |
| 3 Injury type | 8 | 246 |  |  |  |  |  |
| 3.1 BBB scale at 7^th^ day | 8 | 246 |  |  |  |  | *P* = 0.18 |
| 3.1.1 Contusion | 6 | 215 | 1.35 [0.70, 2.00] | < 0.0001 | 94 | < 0.00001 |  |
| 3.1.2 Compression | 2 | 31 | 4.88 [-0.28, 10.05] | 0.06 | 97 | 0.18 |  |
| 3.2 BBB scale at 14^th^ day | 7 | 222 |  |  |  |  | *P* < 0.00001 |
| 3.2.1 Contusion | 5 | 191 | 1.93 [1.82, 2.05] | < 0.00001 | 4 | 0.39 |  |
| 3.2.2 Compression | 2 | 31 | 6.81 [5.41, 8.20] | < 0.00001 | 41 | 0.19 |  |
| 3.3 BBB scale at 21^st^ day | 6 | 182 |  |  |  |  | *P* < 0.00001 |
| 3.3.1 Contusion | 4 | 151 | 2.49 [2.04, 2.93] | < 0.00001 | 45 | 0.12 |  |
| 3.3.2 Compression | 2 | 31 | 7.65 [5.48, 9.82] | < 0.00001 | 66 | 0.08 |  |
| 3.4 BBB scale at 28^th^ day | 7 | 222 |  |  |  |  | *P* = 0.004 |
| 3.4.1 Contusion | 5 | 191 | 3.44 [2.06, 4.81] | < 0.00001 | 97 | < 0.00001 |  |
| 3.4.2 Compression | 2 | 31 | 7.77 [5.20, 10.34] | < 0.00001 | 73 | 0.06 |  |
| 3.5 BBB scale at 35^th^ day | 5 | 172 |  |  |  |  | *P* < 0.0001 |
| 3.5.1 Contusion | 4 | 151 | 3.44 [2.79, 4.09] | < 0.00001 | 73 | 0.005 |  |
| 3.5.2 Compression | 1 | 21 | 9.06 [6.60, 11.52] | < 0.00001 | N/A | N/A |  |
| 3.6 BBB scale at 42^nd^ day | 6 | 212 |  |  |  |  | *P* = 0.0003 |
| 3.6.1 Contusion | 5 | 191 | 3.74 [2.89, 4.59] | < 0.00001 | 92 | < 0.00001 |  |
| 3.6.2 Compression | 1 | 21 | 8.96 [6.27, 11.66] | < 0.00001 | N/A | N/A |  |
| 4 Administration method | 8 | 246 |  |  |  |  |  |
| 4.1 BBB scale at 7^th^ day | 8 | 246 |  |  |  |  | *P* = 0.49 |
| 4.1.1 Intravenous | 5 | 206 | 1.88 [1.07, 2.69] | < 0.00001 | 95 | < 0.00001 |  |
| 4.1.2 Intraperitoneal | 3 | 40 | 2.25 [1.59, 2.90] | < 0.00001 | 42 | 0.18 |  |
| 4.2 BBB scale at 14^th^ day | 7 | 222 |  |  |  |  | *P* = 0.0003 |
| 4.2.1 Intravenous | 5 | 206 | 2.41 [1.80, 3.02] | < 0.00001 | 87 | < 0.00001 |  |
| 4.2.2 Intraperitoneal | 2 | 16 | 5.95 [4.14, 7.76] | < 0.00001 | 17 | 0.27 |  |
| 4.3 BBB scale at 21^st^ day | 6 | 182 |  |  |  |  | *P* = 0.007 |
| 4.3.1 Intravenous | 4 | 166 | 3.17 [2.15, 4.19] | < 0.00001 | 89 | < 0.00001 |  |
| 4.3.2 Intraperitoneal | 2 | 16 | 6.12 [4.26, 7.99] | < 0.00001 | 38 | 0.2 |  |
| 4.4 BBB scale at 28^th^ day | 7 | 222 |  |  |  |  | *P* = 0.21 |
| 4.4.1 Intravenous | 5 | 206 | 4.08 [2.68, 5.48] | < 0.00001 | 97 | < 0.00001 |  |
| 4.4.2 Intraperitoneal | 2 | 16 | 5.77 [3.53, 8.00] | < 0.00001 | 54 | 0.14 |  |
| 4.5 BBB scale at 35^th^ day | 5 | 172 |  |  |  |  | *P* = 0.64 |
| 4.5.1 Intravenous | 4 | 166 | 3.93 [2.94, 4.91] | < 0.00001 | 88 | < 0.00001 |  |
| 4.5.2 Intraperitoneal | 1 | 6 | 4.54 [2.15, 6.93] | 0.0002 |  |  |  |
| 4.6 BBB scale at 42^nd^ day | 6 | 212 |  |  |  |  | *P* = 0.97 |
| 4.6.1 Intravenous | 5 | 206 | 4.11 [3.20, 5.01] | < 0.00001 | 93 | < 0.00001 |  |
| 4.6.2 Intraperitoneal | 1 | 6 | 4.03 [0.73, 7.33] | 0.02 |  |  |  |
| 5 Administration times | 8 | 246 |  |  |  |  |  |
| 5.1 BBB scale at 7^th^ day | 8 | 246 |  |  |  |  | *P* = 0.32 |
| 5.1.1 Multiple injection | 8 | 213 | 1.54 [0.97, 2.10] | < 0.00001 | 92 | < 0.00001 |  |
| 5.1.2 Single injection | 2 | 33 | 4.52 [-1.34, 10.39] | 0.13 | 98 | < 0.00001 |  |
| 5.2 BBB scale at 14^th^ day | 8 | 222 |  |  |  |  | *P* = 0.75 |
| 5.2.1 Multiple injection | 7 | 189 | 2.62 [1.97, 3.27] | < 0.0001 | 89 | < 0.00001 |  |
| 5.2.2 Single injection | 2 | 33 | 3.85 [-3.80, 11.49] | < 0.00001 | 98 | 0.32 |  |
| 5.3 BBB scale at 21^st^ day | 7 | 182 |  |  |  |  | *P* = 0.72 |
| 5.3.1 Multiple injection | 6 | 149 | 3. 16 [2.36, 3.96] | < 0.00001 | 84 | < 0.00001 |  |
| 5.3.2 Single injection | 2 | 33 | 4.70[-3.54, 12.93] | < 0.00001 | 98 | 0.26 |  |
| 5.4 BBB scale at 28^th^ day | 8 | 222 |  |  |  |  | *P* = 0.79 |
| 5.4.1 Multiple injection | 7 | 189 | 3.70 [2.71, 4.69] | < 0.00001 | 96 | < 0.00001 |  |
| 5.4.2 Single injection | 2 | 33 | 4.85 [-3.68, 13.38] | < 0.00001 | 98 | 0.27 |  |
| 5.5 BBB scale at 35^th^ day | 5 | 172 |  |  |  |  | *P* = 0.82 |
| 5.5.1 Multiple injection | 4 | 139 | 3.60 [3.10, 4.11] | < 0.00001 | 66 | 0.08 |  |
| 5.5.2 Single injection | 2 | 33 | 4.62 [-3.93, 13.17] | 0.29 | 98 | < 0.00001 |  |
| 5.6 BBB scale at 42^nd^ day | 6 | 212 |  |  |  |  | *P* = 0.80 |
| 5.6.1 Multiple injection | 5 | 179 | 3.83 [3.17, 4.50] | < 0.00001 | 88 | < 0.00001 |  |
| 5.6.2 Single injection | 2 | 33 | 4.85 [-3.00, 12.70] | 0.23 | 97 | < 0.00001 |  |
| 6 Administration dose | 8 | 252 |  |  |  |  |  |
| 6.1 BBB scale at 7^th^ day | 7 | 222 |  |  |  |  | *P* = 0.15 |
| 6.1.1 ˂ 5 mg/(kg·d) | 2 | 63 | 1.02 [0.39, 1.65] | 0.002 | 70 | 0.03 |  |
| 6.1.2 5-10 mg/(kg·d) | 6 | 101 | 1.69 [0.83, 2.55] | 0.0001 | 81 | < 0.00001 |  |
| 6.1.3 >10 mg/(kg·d) | 2 | 58 | 2.29 [1.06, 3.51] | 0.0003 | 86 | < 0.0001 |  |
| 6.2 BBB scale at 14^th^ day | 7 | 222 |  |  |  |  | *P* = 0.08 |
| 6.2.1 ˂ 5 mg/(kg·d) | 2 | 63 | 1.35 [0.11, 2.59] | 0.03 | 92 | < 0.0001 |  |
| 6.2.2 5-10 mg/(kg·d) | 6 | 101 | 3.39 [2.02, 4.76] | < 0.00001 | 90 | < 0.00001 |  |
| 6.2.3 >10 mg/(kg·d) | 2 | 58 | 2.71 [1.80, 3.61] | < 0.00001 | 74 | 0.004 |  |
| 6.3 BBB scale at 21^st^ day | 6 | 182 |  |  |  |  | *P* = 0.14 |
| 5.3.1 ˂ 5 mg/(kg·d) | 1 | 23 | 1.51 [-0.47, 3.48] | 0.13 | 86 | 0.008 |  |
| 5.3.2 5-10 mg/(kg·d) | 6 | 101 | 3.85 [2.59, 5.10] | < 0.00001 | 88 | < 0.00001 |  |
| 5.3.3 >10 mg/(kg·d) | 2 | 58 | 2.95 [1.71, 4.18] | < 0.00001 | 79 | 0.0009 |  |
| 6.4 BBB scale at 28^th^ day | 7 | 222 |  |  |  |  | *P* = 0.38 |
| 6.4.1 ˂ 5 mg/(kg·d) | 2 | 63 | 2.84[-0.18, 5.87] | < 0.00001 | 98 | 0.07 |  |
| 6.4.2 5-10 mg/(kg·d) | 6 | 101 | 4.09 [2.99, 5.19] | < 0.00001 | 82 | 0.001 |  |
| 6.4.3 >10 mg/(kg·d) | 2 | 58 | 3.11 [2.14, 4.08] | < 0.00001 | 77 | 0.001 |  |
| 6.5 BBB scale at 35^th^ day | 5 | 226 |  |  |  |  | *P* = 0.72 |
| 6.5.1 ˂ 5 mg/(kg·d) | 2 | 77 | 2.68 [0.13, 5.23] | < 0.00001 | 95 | 0.04 |  |
| 6.5.2 5-10 mg/(kg·d) | 5 | 91 | 3.77 [2.97, 4.56] | < 0.00001 | 72 | 0.001 |  |
| 6.5.3 >10 mg/(kg·d) | 2 | 58 | 3.75 [2.65, 4.85] | < 0.00001 | 79 | 0.0009 |  |
| 6.6 BBB scale at 42^nd^ day | 6 | 252 |  |  |  |  | *P* = 0.76 |
| 6.6.1 ˂ 5 mg/(kg·d) | 2 | 53 | 2.87 [0.31, 5.44] | < 0.00001 | 97 | 0.03 |  |
| 6.6.2 5-10 mg/(kg·d) | 6 | 131 | 3.90 [3.10, 4.69] | < 0.00001 | 84 | < 0.00001 |  |
| 6.6.3 >10 mg/(kg·d) | 2 | 58 | 3.82 [2.63, 5.01] | < 0.00001 | 77 | 0.001 |  |

**Table S2. Summary of sensitivity analysis.**

| **BBB scale** | **Studies exclusion** | **No. of animals** | **Weighted mean difference** | | **Heterogeneity** | |
| --- | --- | --- | --- | --- | --- | --- |
|  |  |  | **95% CI** | ***P* value** | ***I*^2^** | ***P* value** |
| 1 BBB scale at 7^th^ day | All included | 246 | 1.96 [1.23, 2.68] | < 0.00001 | 95 | < 0.00001 |
|  | Without outcome assessor blinding | 201 | 1.29 [0.75, 1.82] | < 0.00001 | 88 | < 0.00001 |
|  | Small sample studies | 230 | 2.02 [1.21, 2.83] | < 0.00001 | 96 | < 0.00001 |
|  | Ishii H et al. 2018 | 225 | 1.47 [0.85, 2.09] | < 0.00001 | 93 | < 0.00001 |
|  | Li RB et al. 2018 | 222 | 1.84 [1.12, 2.57] | < 0.00001 | 94 | < 0.00001 |
|  | Ohta S et al. 2005 | 227 | 2.00 [1.25, 2.76] | < 0.00001 | 96 | < 0.00001 |
|  | Ohta S et al. 2011 1 | 187 | 2.14 [1.29, 3.00] | < 0.00001 | 96 | < 0.00001 |
|  | Ohta S et al. 2011 3 | 199 | 2.01 [1.17, 2.85] | < 0.00001 | 95 | < 0.00001 |
|  | Pang YL et al. 2022 | 240 | 2.06 [1.30, 2.82] | < 0.00001 | 96 | < 0.00001 |
|  | Ren XS et al. 2006 | 226 | 2.06 [1.21, 2.92] | < 0.00001 | 96 | < 0.00001 |
|  | Song YY et al. 2015 | 206 | 2.17 [1.38, 2.96] | < 0.00001 | 91 | < 0.00001 |
|  | Xu B et al. 2023 | 236 | 1.91 [1.14, 2.68] | < 0.00001 | 96 | < 0.00001 |
| 2 BBB scale at 14^th^ day | All included | 222 | 3.15 [2.31, 3.99] | < 0.00001 | 93 | < 0.00001 |
|  | Without outcome assessor blinding | 201 | 2.66 [1.92, 3.40] | < 0.00001 | 91 | < 0.00001 |
|  | Small sample studies | 206 | 2.41 [1.80, 3.02] | < 0.00001 | 87 | < 0.00001 |
|  | Ishii H et al. 2018 | 201 | 2.66 [1.92, 3.40] | < 0.00001 | 91 | < 0.00001 |
|  | Ohta S et al. 2005 | 203 | 3.25 [2.36, 4.14] | < 0.00001 | 94 | < 0.00001 |
|  | Ohta S et al. 2011 1 | 163 | 3.42 [2.31, 4.53] | < 0.00001 | 94 | < 0.00001 |
|  | Ohta S et al. 2011 3 | 175 | 3.51 [2.47, 4.55] | < 0.00001 | 94 | < 0.00001 |
|  | Pang YL et al. 2022 | 216 | 3.14 [2.29, 3.99] | < 0.00001 | 94 | < 0.00001 |
|  | Ren XS et al. 2006 | 202 | 3.38 [2.37, 4.39] | < 0.00001 | 94 | < 0.00001 |
|  | Song YY et al. 2015 | 182 | 3.56 [2.25, 4.86] | < 0.00001 | 93 | < 0.00001 |
|  | Xu B et al. 2023 | 212 | 2.42 [1.82, 3.02] | < 0.00001 | 84 | < 0.00001 |
| 3 BBB scale at 21^th^ day | All included | 182 | 3.97 [2.80, 5.15] | < 0.00001 | 92 | < 0.00001 |
|  | Without outcome assessor blinding | 161 | 3.29 [2.28, 4.31] | < 0.00001 | 90 | < 0.00001 |
|  | Small sample studies | 166 | 3.17 [2.15, 4.19] | < 0.00001 | 89 | < 0.00001 |
|  | Ishii H et al. 2018 | 161 | 3.29 [2.28, 4.31] | < 0.00001 | 90 | < 0.00001 |
|  | Ohta S et al. 2005 | 163 | 4.20 [2.94, 5.46] | < 0.00001 | 94 | < 0.00001 |
|  | Ohta S et al. 2011 1 | 123 | 4.39 [2.90, 5.89] | < 0.00001 | 93 | < 0.00001 |
|  | Ohta S et al. 2011 3 | 135 | 4.46 [2.91, 6.01] | < 0.00001 | 93 | < 0.00001 |
|  | Pang YL et al. 2022 | 176 | 3.92 [2.69, 5.15] | < 0.00001 | 93 | < 0.00001 |
|  | Ren XS et al. 2006 | 162 | 4.37 [2.57, 6.16] | < 0.00001 | 94 | < 0.00001 |
|  | Xu B et al. 2023 | 172 | 3.28 [2.30, 4.26] | < 0.00001 | 87 | < 0.00001 |
| 4 BBB scale at 28^th^ day | All included | 222 | 4.41 [3.19, 5.63] | < 0.00001 | 97 | < 0.00001 |
|  | Without outcome assessor blinding | 201 | 3.89 [2.63, 5.15] | < 0.00001 | 97 | < 0.0003 |
|  | Small sample studies | 206 | 4.08 [2.68, 5.48] | < 0.00001 | 97 | < 0.00001 |
|  | Ishii H et al. 2018 | 201 | 3.89 [2.63, 5.15] | < 0.00001 | 97 | < 0.0003 |
|  | Ohta S et al. 2005 | 203 | 4.56 [3.26, 5.85] | < 0.00001 | 97 | < 0.00001 |
|  | Ohta S et al. 2011 1 | 163 | 4.70 [3.37, 6.03] | < 0.00001 | 96 | < 0.00001 |
|  | Ohta S et al. 2011 3 | 175 | 4.74 [3.56, 5.93] | < 0.00001 | 94 | < 0.00001 |
|  | Pang YL et al. 2022 | 216 | 4.43 [3.15, 5.72] | < 0.00001 | 97 | < 0.00001 |
|  | Ren XS et al. 2006 | 202 | 4.61 [3.19, 6.03] | < 0.00001 | 97 | < 0.00001 |
|  | Song YY et al. 2015 | 182 | 4.26 [3.05, 5.46] | < 0.00001 | 91 | < 0.00001 |
|  | Xu B et al. 2023 | 212 | 4.09 [2.77, 5.42] | < 0.00001 | 97 | < 0.00001 |
| 5 BBB scale at 35^th^ day | All included | 172 | 3.97 [3.05, 4.89] | < 0.00001 | 86 | < 0.00001 |
|  | Without outcome assessor blinding | 151 | 3.44 [2.79, 4.09] | < 0.00001 | 73 | =0.005 |
|  | Small sample studies | 166 | 3.93 [2.94, 4.91] | < 0.00001 | 88 | < 0.00001 |
|  | Ishii H et al. 2018 | 151 | 3.44 [2.79, 4.09] | < 0.00001 | 73 | =0.005 |
|  | Ohta S et al. 2005 | 153 | 4.21 [3.03, 5.40] | < 0.00001 | 89 | < 0.00001 |
|  | Ohta S et al. 2011 1 | 113 | 4.33 [3.13, 5.53] | < 0.00001 | 87 | < 0.00001 |
|  | Ohta S et al. 2011 3 | 125 | 4.34 [3.23, 5.45] | < 0.00001 | 86 | < 0.00001 |
|  | Pang YL et al. 2022 | 166 | 3.93 [2.94, 4.91] | < 0.00001 | 88 | < 0.00001 |
|  | Ren XS et al. 2006 | 152 | 3.91 [2.85, 4.98] | < 0.00001 | 84 | < 0.0001 |
| 6 BBB scale at 42^th^ day | All included | 212 | 4.10 [3.23, 4.97] | < 0.00001 | 91 | < 0.00001 |
|  | Without outcome assessor blinding | 191 | 3.74 [2.89, 4.59] | < 0.00001 | 92 | < 0.00001 |
|  | Small sample studies | 206 | 4.11 [3.20, 5.01] | < 0.00001 | 93 | < 0.00001 |
|  | Ishii H et al. 2018 | 191 | 3.74 [2.89, 4.59] | < 0.00001 | 92 | < 0.00001 |
|  | Ohta S et al. 2005 | 193 | 4.27 [3.29, 5.25] | < 0.00001 | 92 | < 0.00001 |
|  | Ohta S et al. 2011 1 | 153 | 4.33 [3.44, 5.22] | < 0.00001 | 88 | < 0.00001 |
|  | Ohta S et al. 2011 3 | 165 | 4.32 [3.37, 5.26] | < 0.00001 | 90 | < 0.00001 |
|  | Pang YL et al. 2022 | 206 | 4.11 [3.20, 5.01] | < 0.00001 | 93 | < 0.00001 |
|  | Ren XS et al. 2006 | 192 | 4.00 [2.96, 5.05] | < 0.00001 | 93 | < 0.00001 |
|  | Song YY et al. 2015 | 172 | 4.01 [2.99, 5.02] | < 0.00001 | 85 | < 0.00001 |

**Appendix 1: Search strategy for databases**

**Pubmed**

**#1:** edaravone[MeSH Terms] OR edaravone[All Fields] OR norantipyrine[All Fields] OR norphenazone[All Fields] OR edarabone[All Fields] OR 1-Phenyl-3-methyl-5-pyrazolone[All Fields] OR 1 Phenyl 3 methyl 5 pyrazolone[All Fields] OR 3-Methyl-1-phenyl-2-pyrazolin-5-one[All Fields] OR 3 Methyl 1 phenyl 2 pyrazolin 5 one[All Fields] OR MCI 186[All Fields] OR MCI-186[All Fields] OR MCI186[All Fields] OR radicava[All Fields] OR phenylmethylpyrazolone[All Fields]

**#2****:** spinal cord injuries[MeSH Terms] OR trauma, nervous system[MeSH Terms] OR spinal cord diseases[MeSH Terms] OR central cord syndrome[MeSH Terms] OR spinal cord compression[MeSH Terms] OR nervous system diseases[MeSH Terms] OR spinal cord trauma[All Fields] OR cord trauma, spinal[All Fields] OR cord traumas, spinal[All Fields] OR trauma, spinal cord[All Fields] OR traumas, spinal cord[All Fields] OR spinal cord traumas[All Fields] OR myelopathy, traumatic[All Fields] OR myelopathies, traumatic[All Fields] OR traumatic myelopathies[All Fields] OR traumatic myelopathy[All Fields] OR spinal cord injury[All Fields] OR cord injury, spinal[All Fields] OR cord injuries, spinal[All Fields] OR injury, spinal cord[All Fields] OR injuries, spinal cord[All Fields] OR spinal cord injuries[All Fields] OR spinal cord contusion[All Fields] OR cord contusion, spinal[All Fields] OR cord contusions, spinal[All Fields] OR contusion, spinal cord[All Fields] OR contusions, spinal cord[All Fields] OR spinal cord contusions[All Fields] OR spinal cord compression[All Fields] OR cord compression, spinal[All Fields] OR cord compressions, spinal[All Fields] OR compression, spinal cord[All Fields] OR compressions, spinal cord[All Fields] OR spinal cord compressions[All Fields] OR dorsal column injury[All Fields] OR corticospinal tract injury[All Fields] OR nervous system diseases[All Fields] OR nervous system disease[All Fields]

**#3:** rats[MeSH Terms] OR murinae[MeSH Terms] OR rats, inbred strains[MeSH Terms] OR rats[All Fields] OR rat[All Fields] OR rattus[All Fields] OR rats, laboratory[All Fields] OR rat, laboratory[All Fields] OR laboratory rats[All Fields] OR laboratory rat[All Fields]

**#4:** **#1 AND #2 AND #3**

**Web of Science**

**#1: TS =** edaravone OR norantipyrine OR norphenazone OR edarabone OR "1-Phenyl-3-methyl-5-pyrazolone" OR 1 Phenyl 3 methyl 5 pyrazolone OR "3-Methyl-1-phenyl-2-pyrazolin-5-one" OR 3 Methyl 1 phenyl 2 pyrazolin 5 one OR MCI 186 OR MCI-186 OR MCI186 OR radicava OR phenylmethylpyrazolone

**#2: TS =** spinal cord injuries OR trauma, nervous system OR spinal cord diseases OR central cord syndrome OR spinal cord compression OR nervous system diseases OR spinal cord trauma OR cord trauma, spinal OR cord traumas, spinal OR trauma, spinal cord OR traumas, spinal cord OR spinal cord traumas OR myelopathy, traumatic OR myelopathies, traumatic OR traumatic myelopathies OR traumatic myelopathy OR spinal cord injury OR cord injury, spinal OR cord injuries, spinal OR injury, spinal cord OR injuries, spinal cord OR spinal cord contusion OR cord contusion, spinal OR cord contusions, spinal OR contusion, spinal cord OR contusions, spinal cord OR spinal cord contusions OR cord compression, spinal OR cord compressions, spinal OR compression, spinal cord OR compressions, spinal cord OR spinal cord compressions OR dorsal column injury OR corticospinal tract injury OR nervous system disease

**#3: TS =** rats OR murinae OR rats, inbred strains OR rat OR rattus OR rats, laboratory OR rat, laboratory OR laboratory rats OR laboratory rat

**#4=#1 AND #2 AND #3**

**EMBASE：**

**#1:** edaravone OR norantipyrine OR norphenazone OR edarabone OR ‘1-Phenyl-3-methyl-5-pyrazolone’ OR ‘1 Phenyl 3 methyl 5 pyrazolone’ OR ‘3-Methyl-1-phenyl-2-pyrazolin-5-one’ OR ‘3 Methyl 1 phenyl 2 pyrazolin 5 one’ OR ‘MCI 186’ OR ‘MCI-186’ OR MCI186 OR radicava OR phenylmethylpyrazolone

**#2:** ‘spinal cord injuries’ OR ‘trauma, nervous system’ OR ‘spinal cord diseases’ OR ‘central cord syndrome’ OR ‘spinal cord compression’ OR ‘nervous system diseases’ OR ‘spinal cord trauma’ OR ‘cord trauma, spinal’ OR ‘cord traumas, spinal’ OR ‘trauma, spinal cord’ OR ‘traumas, spinal cord’ OR ‘spinal cord traumas’ OR ‘myelopathy, traumatic’ OR ‘myelopathies, traumatic’ OR ‘traumatic myelopathies’ OR ‘traumatic myelopathy’ OR ‘spinal cord injury’ OR ‘cord injury, spinal’ OR ‘cord injuries, spinal’ OR ‘injury, spinal cord’ OR ‘injuries, spinal cord’ OR ‘spinal cord contusion’ OR ‘cord contusion, spinal’ OR ‘cord contusions, spinal’ OR ‘contusion, spinal cord’ OR ‘contusions, spinal cord’ OR ‘spinal cord contusions’ OR ‘spinal cord compression’ OR ‘cord compression, spinal’ OR ‘cord compressions, spinal’ OR ‘compression, spinal cord’ OR ‘compressions, spinal cord’ OR ‘spinal cord compressions’ OR ‘dorsal column injury’ OR ‘corticospinal tract injury’ OR ‘nervous system disease’

**#3:**  rats OR murinae OR ‘rats, inbred strains’ OR rats OR rat OR rattus OR ‘rats, laboratory’ OR ‘rat, laboratory’ OR ‘laboratory rats’ OR ‘laboratory rat’

**#4=#1 AND #2 AND #3**

**Scopus**

**#1: TITLE-ABS-KEY** (edaravone) OR **(**norantipyrine) OR **(**norphenazone) OR **(**edarabone) OR **(**"1-Phenyl-3-methyl-5-pyrazolone") OR **(**"1 Phenyl 3 methyl 5 pyrazolone") OR **(**"3-Methyl-1-phenyl-2-pyrazolin-5-one") OR **(**"3 Methyl 1 phenyl 2 pyrazolin 5 one") OR **(**"MCI 186") OR **(**"MCI-186") OR **(**MCI186) OR **(**radicava) OR (phenylmethylpyrazolone)

**#2: TITLE-ABS-KEY** ("spinal cord injuries") OR **(**"trauma, nervous system") OR **(**"spinal cord diseases") OR **(**"central cord syndrome") OR **(**"spinal cord compression") OR **(**"nervous system diseases") OR **(**"spinal cord trauma") OR **(**"cord trauma, spinal") OR **(**"cord traumas, spinal") OR **(**"trauma, spinal cord") OR **(**"traumas, spinal cord") OR **(**"spinal cord traumas") OR **(**"myelopathy, traumatic") OR **(**"myelopathies, traumatic") OR **(**"traumatic myelopathies") OR **(**"traumatic myelopathy") OR **(**"spinal cord injury") OR **(**"cord injury, spinal") OR **(**"cord injuries, spinal") OR **(**"injury, spinal cord") OR **(**"injuries, spinal cord") OR **(**"spinal cord contusion") OR **(**"cord contusion, spinal") OR **(**"cord contusions, spinal") OR **(**"contusion, spinal cord") OR **(**"contusions, spinal cord") OR **(**"spinal cord contusions") OR **(**"cord compression, spinal") OR **(**"cord compressions, spinal") OR **(**"compression, spinal cord") OR **(**"compressions, spinal cord") OR **(**"spinal cord compressions") OR **(**"dorsal column injury") OR **(**"corticospinal tract injury") OR **(**"nervous system disease")

**#3: TITLE-ABS-KEY** (rats) OR **(**murinae) OR **(**"rats, inbred strains") OR **(**rat) OR **(**rattus) OR **(**rats, "laboratory") OR **(**"rat, laboratory") OR **(**"laboratory rats") OR **(**"laboratory rat")

**#4=#1 AND #2 AND #3**

**Cochrance：**

**#1:** MeSH descriptor edaravone explode all trees OR edaravone OR norantipyrine OR norphenazone OR edarabone OR "1-Phenyl-3-methyl-5-pyrazolone" OR 1 Phenyl 3 methyl 5 pyrazolone OR "3-Methyl-1-phenyl-2-pyrazolin-5-one" OR 3 Methyl 1 phenyl 2 pyrazolin 5 one OR MCI 186 OR MCI-186 OR MCI186 OR radicava OR phenylmethylpyrazolone

**#2:** MeSH descriptor spinal cord injuries explode all trees OR MeSH descriptor trauma, nervous system explode all trees OR MeSH descriptor spinal cord diseases explode all trees OR MeSH descriptor central cord syndrome explode all trees OR MeSH descriptor spinal cord compression explode all trees OR MeSH descriptor nervous system diseases explode all trees OR spinal cord trauma OR cord trauma, spinal OR cord traumas, spinal OR trauma, spinal cord OR traumas, spinal cord OR spinal cord traumas OR myelopathy, traumatic OR myelopathies, traumatic OR traumatic myelopathies OR traumatic myelopathy OR spinal cord injury OR cord injury, spinal OR cord injuries, spinal OR injury, spinal cord OR injuries, spinal cord OR spinal cord injuries OR spinal cord contusion OR cord contusion, spinal OR cord contusions, spinal OR contusion, spinal cord OR contusions, spinal cord OR spinal cord contusions OR spinal cord compression OR cord compression, spinal OR cord compressions, spinal OR compression, spinal cord OR compressions, spinal cord OR spinal cord compressions OR dorsal column injury OR corticospinal tract injury OR nervous system diseases OR nervous system disease

**#3:** MeSH descriptor rats explode all trees OR MeSH descriptor murinae explode all trees OR MeSH descriptor rats, inbred strains explode all trees OR rats OR rat OR rattus OR rats, laboratory OR rat, laboratory OR laboratory rats

**#4=#1 AND #2 AND #3**
